# Structure-oriented substrate specificity engineering of aldehyde-deformylating oxygenase towards aldehydes carbon chain length

**DOI:** 10.1186/s13068-016-0596-9

**Published:** 2016-08-31

**Authors:** Luyao Bao, Jian-Jun Li, Chenjun Jia, Mei Li, Xuefeng Lu

**Affiliations:** 1 Key Laboratory of Biofuels, Chinese Academy of Sciences, Qingdao, China; 2 Shandong Provincial Key Laboratory of Synthetic Biology, Qingdao Institute of Bioenergy and Bioprocess Technology, Chinese Academy of Sciences, No. 189 Songling Road, Qingdao, 266101 China; 3 University of Chinese Academy of Sciences, Beijing, 100049 China; 4 National Laboratory of Biomacromolecules, Institute of Biophysics, Chinese Academy of Sciences, 15 Datun Road, Chaoyang District, Beijing, 100101 China; 5 National Key Laboratory of Biochemical Engineering, Institute of Process Engineering, Chinese Academy of Sciences, 1 North 2nd Street, Haidian District, Beijing, 100190 China

**Keywords:** Aldehyde-deformylating oxygenase, Site-directed mutagenesis, Structure-guided protein engineering, Chain-length selectivity, *Synechococcus elongatus* PCC7942

## Abstract

**Background:**

Aldehyde-deformylating oxygenase (ADO) is an important enzyme involved in the biosynthetic pathway of fatty alk(a/e)nes in cyanobacteria. However, ADO exhibits quite low chain-length specificity with respect to the substrates ranging from C_4_ to C_18_ aldehydes, which is not suitable for producing fuels with different properties or different chain lengths.

**Results:**

Based on the crystal structures of cADOs (cyanobacterial ADO) with substrate analogs bound, some amino acids affecting the substrate specificity of cADO were identified, including the amino acids close to the aldehyde group and the hydrophobic tail of the substrate and those along the substrate channel. Using site-directed mutagenesis, selected amino acids were replaced with bulky ones introducing steric hindrance to the binding pocket via large functional groups. All mutants were overexpressed, purified and kinetically characterized. All mutants, except F87Y, displayed dramatically reduced activity towards C_14,16,18_ aldehydes. Notably, the substrate preferences of some mutants towards different chain-length substrates were enhanced: I24Y for *n*-heptanal, I27F for *n*-decanal and *n*-dodecanal, V28F for *n*-dodecanal, F87Y for *n*-decanal, C70F for *n*-hexanal, A118F for *n*-butanal, A121F for C_4,6,7_ aldehydes, V184F for *n*-dodecanal and *n*-decanal, M193Y for C_6–10_ aldehydes and L198F for C_7–10_ aldehydes. The impact of the engineered cADO mutants on the change of the hydrocarbon profile was demonstrated by co-expressing acyl-ACP thioesterase *BTE*, *fadD* and V184F in *E. coli*, showing that *n*-undecane was the main fatty alkane.

**Conclusions:**

Some amino acids, which can control the chain-length selectivity of substrates of cADO, were identified. The substrate specificities of cADO were successfully changed through structure-guided protein engineering, and some mutants displayed different chain-length preference. The in vivo experiments of V184F in genetically engineered *E. coli* proved the importance of engineered cADOs on the distribution of the fatty alkane profile. The results would be helpful for the production of fatty alk(a/e)nes in cyanobacteria with different properties.

**Electronic supplementary material:**

The online version of this article (doi:10.1186/s13068-016-0596-9) contains supplementary material, which is available to authorized users.

## Background

The biosynthesis of fatty alk(a/e)nes by plants, insects, birds, green algae and cyanobacteria has been attracting great attention, since fatty alk(a/e)nes have been considered as the ideal replacements for fossil-based fuels [1–5]. It has been accepted that one of the enzymatic pathways producing alk(a/e)nes is derived from fatty acyl-ACP or -CoA in a two-step reaction: fatty acyl-ACP or -CoA is first reduced into fatty aldehyde by acyl-ACP or -CoA reductase, then fatty aldehyde is converted into alk(a/e)ne by aldehyde decarbonylase (now renamed as aldehyde-deformylating oxygenase, ADO). In 2010, Schirmer et al. identified two genes involved in alk(a/e)ne biosynthesis in cyanobacteria: acyl-ACP reductase and ADO [1]. In 2013, Akhtar et al. reported that a carboxylic acid reductase (CAR) from *Mycobacterium marinum* could convert a wide range of aliphatic fatty acids (C_6_–C_18_) into corresponding aldehydes, which can then be transformed into fatty alkane by ADO [6]. From the viewpoint of chemistry, transformation of aldehydes into alk(a/e)nes by ADO is quite difficult and unusual, so cADO (cyanobacterial ADO) has attracted particular interest in industry and academia [7].

**Scheme 1:**
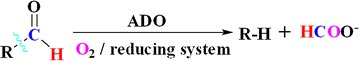
cADO-catalyzed reaction [8–10]

Since then, several important conclusions have been drawn: (1) the C1-derived coproduct of the cADO-catalyzed reaction is formate, instead of previously supposed carbon monoxide [8]; (2) oxygen is absolutely required, and one O-atom is incorporated into formate [9, 10]; (3) the auxiliary reducing system providing four electrons is needed, and the homologous electron transfer system worked more effectively than the heterologous and chemical ones in supporting cADO activity [1, 9, 11–13] (Scheme 1). It has been observed that self-sufficient cADOs fused to homogenous ferredoxin (Fd) and ferredoxin-NADP^+^ reductase (FNR) could efficiently catalyze conversion of aldehydes into alk(a/e)ne [14]. Andre et al. reported that cADO was reversibly inhibited by H_2_O_2_ originating from poor coupling of reductant consumption with alk(a/e)ne formation, and the kinetics of cADO towards aldehyde substrates of carbon chain lengths between 8 and 18 carbons showed that cADO did not exhibit strong chain-length specificity with respect to its substrates [15]. cADO also produces *n*-1 aldehydes and alcohols in addition to alk(a/e)ne [16]. Mechanistic studies have demonstrated that a radical intermediate is involved in the cADO-catalyzed reaction, and a possible catalytic process has been proposed based on the crystal structures of cADO from *Synechococcus elongatus* strain PCC7942 [17–20]. cADO was engineered to improve specificity for short- to medium-chain aldehydes [21]. Hayashi et al. investigated the role of three cysteines in the structure, stability and alk(a/e)ne production of cADO [22]. Based on the crystal structures of cADO, cADO belongs to the non-heme dinuclear iron oxygenase family of enzymes including methane monoxygenase, type I ribonucleotide reductase and ferritin [1, 17, 23–25].

Fatty alk(a/e)nes are the main component of traditional fuels such as gasoline, diesel and jet fuel. The carbon number distribution of hydrocarbons varies in different fuels, for example, 4–12 in gasoline, 9–23 in diesel and 8–16 in jet fuel [26]. Increasing interest in developing the next generation of biofuels, which can function as “drop-in” fuels, has spurred high attention towards the enzymes involved in hydrocarbon biosynthesis. The acyl-ACP thioesterases with different carbon chain-length specificity could be used to synthesize the fatty acid-based fuels such as fatty alcohols and FAEs (fatty acid esters) with different carbon chain length distributions [27]. A number of different carbon chain length-specific acyl-ACP thioesterases have been successfully utilized to control the carbon chain length distributions of fatty acids and/or fatty acid derivatives in genetically engineered microbes, such as *tesA* from *Escherichia coli* (C16:0), *CCTE* from *Cinnamomum camphora* (C14:0), and *BTE* from *Umbellularia californica* (C12:0) [28]. Moreover, engineering efforts have also been successful in altering the specificity of wild-type desaturases, such as the Castor Δ^9^-18:0-ACP desaturase, leading to the isolation of mutants with up to 15-fold increased specific activity towards 16-carbon substrates [29]. The substrate specificity of β-ketoacyl-ACP synthase was modified from 8:0-ACP substrate to 6:0-ACP through protein engineering [30]. Very recently, an acyl carrier protein (ACP) from *Synechococcus elongatus*was engineered to enhance production of shortened fatty acids such as C_14_ fatty acid [31].

Alternatively, structure-orientated substrate specificity engineering of cADO would also facilitate production of these “drop in” biofuels with different carbon chain-length distributions. However, it was observed that cADO-PMT1231 from *Prochlorococcus marinus* (strain MIT9313) exhibits quite low chain-length specificity (C_4–18_) with respect to the aldehyde substrates [15]. Given that all cADOs showed similar structural characteristics (structural superimposition of cADO-1593 and cADO-PMT1231) (Additional file 1: Figure S1), the cADO enzymes from different cyanobacterial species might have similar substrate profiles. Therefore, the available crystal structures of cADOs with the bound substrate analogs such as fatty acids or fatty alcohols or fatty aldehydes have enabled structure-guided substrate specificity engineering of cADOs [1, 17, 21, 23].

In this current study, the amino acids of cADO-1593 from *Synechococcus elongatus* which might influence the chain-length selectivity of the substrates were identified and fully characterized. The substrate specificities of cADO towards different chain-length substrates were achieved through structure-orientated engineering, and in vivo experiments were also performed by introducing an engineered ADO mutant into a fatty alk(a/e)ne producing *E. coli* factory.

## Results

### Identification of the amino acids that may influence the substrate specificity of cADO

**Fig. 1:**
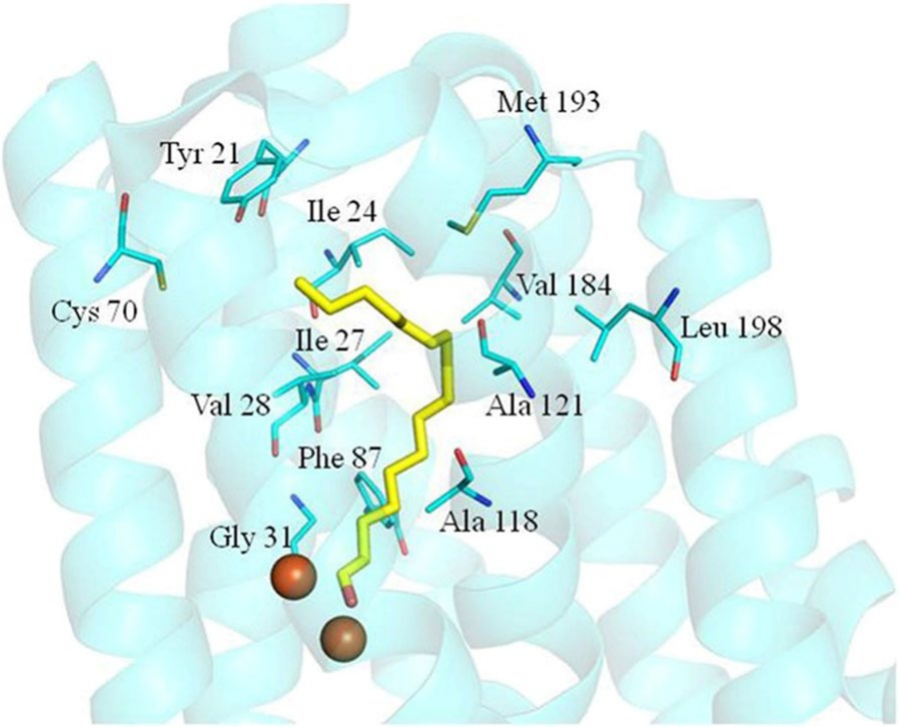
Identified residues which might affect the substrate specificity of cADO (PDB code: 4RC5). The identified residues include those close to the aldehyde group (Gly31 and Ala118) and the hydrophobic tail (Tyr21 and Cys70) of the substrate, and those along the substrate channel (Ile24, Ile27, Val28, Phe87, Ala121, Val184, Met193 and Leu198). The bound substrate analog was colored *yellow*

According to the crystal structure of cADO-1593 from *Synechococcus elongatus* strain PCC7942 (PDB code: 4RC5) [17], amino acids involved in the substrate channel were identified, including Tyr21, Ile24, Ile27, Val28, Gly31, Phe67, Cys70, Phe86, Phe87, Phe117, Ala118, A121, Tyr122, Tyr125, Val184, Met193 and Leu198 (Fig. 1). Since the side chains of Phe67, Phe86, Phe117, Tyr122 and Tyr125 are either parallel with the substrate analog or do not point towards the substrate analog and only provide a hydrophobic environment for the substrate, they are not investigated in the current study. The other amino acids, which might have some influence on the substrate specificity of cADO, were investigated.

Investigation into the substrate tunnel of cADO-1593 revealed that the amino acids, Gly31 and Ala118 are close to the di-iron center and the aldehyde group of the substrate (<C_4_). Replacement of these two amino acids with tyrosine or phenylalanine, which may introduce a steric block in this position, might improve the selectivity of cADO for short-chain substrates against medium- and long-chain substrates.

Amino acids including Ile24, Ile27, Val28, Phe87, Ala121, Val184 and Leu198 were also identified along the substrate tunnel. All of them point their side chains towards the bound substrates and their side chains are approximately perpendicular to the substrate. Both Val41 and Ala134 of cADO PMT1231 from *Prochlorococcus marinus* (MIT9313) [21] have been shown to have effects on substrate specificity, which are, respectively equivalent to the sites of Val28 and Ala121 of cADO-1593. These amino acids might also have some impact on access of medium and long-chain length substrates when they are replaced with bulky ones, such as tyrosine or phenylalanine.

The amino acids Tyr21 and Cys70 are situated close to the hydrophobic end of the substrate analog according to the crystal structure of cADO-1593, which was predicted to be the possible substrate entrance. Therefore, mutation of them into the bulky ones might have some influence on substrate entry.

### Site-directed mutagenesis, overexpression, purification and enzymatic assays of WT and cADO mutants

Considering that replacement of the above identified amino acids with large ones might impede the binding of substrates beyond certain length or substrate access, thirteen mutants, including Y21R, I24Y, I27F, V28Y, G31F, C70F, F87Y, A118F, A121F, V184F, M193Y and L198F, were constructed following the standard protocol using WT cADO-1593 as the template. All mutants were successfully overexpressed in *E. coli* BL21(DE3) and purified on a nickel column as previous described [13] (Additional file 2: Figure S2). All enzymatic assays were carried out in the presence of the chemical reducing system NADH/PMS (phenazine methosulfate) and catalase [13].

### Activities towards medium-to long-chain length aldehydes

**Fig. 2:**
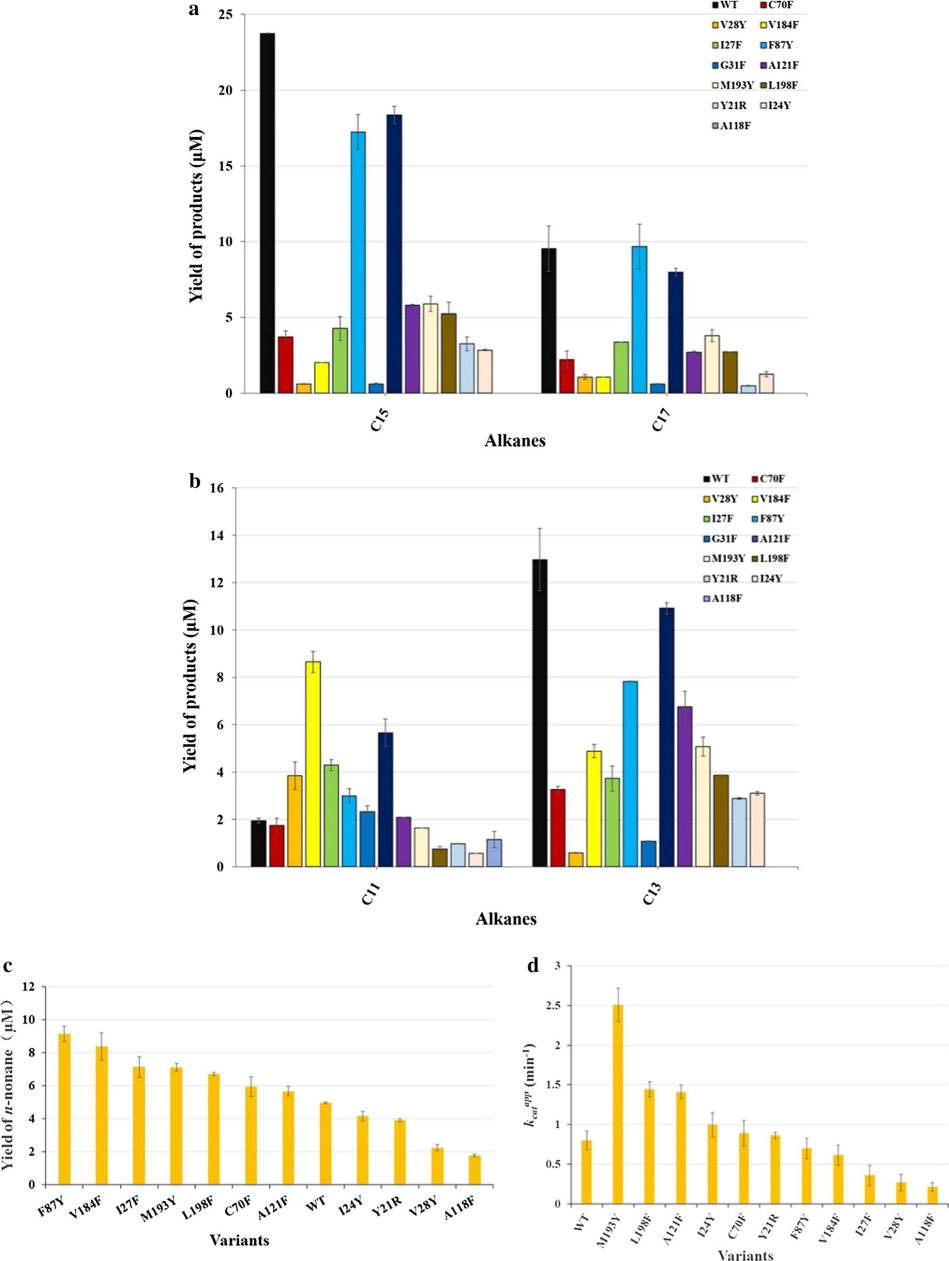
Yields of fatty alkanes for wild-type cADO and variants towards C_10,12,14,16,18_ aldehydes (**a**– **c)** and the apparent *k*_*cat*_ values towards *n*-heptanal (**d**). Yields of fatty alkanes for wild-type cADO and variants towards C_10_,_12,14,16,18_ aldehydes were determined by GC–MS and using C_20_ alkane (10 μM) as an internal standard. The amount of *n*-hexane produced was quantified by GC and a standard curve of known concentrations of *n*-hexane

Medium- and long-chain (C_10,12,14,16,18_) aldehydes were used as the substrates to investigate the effects of the mutations on the substrate specificity (Fig. 2; Additional file 3). A118F did not show any obvious activity against C_14,16,18_ aldehydes, and only exhibited slight activity towards *n*-dodecanal and *n*-decanal. Eleven mutants, excluding F87Y, displayed dramatically reduced activity towards C_14,16,18_ aldehydes. Notably, the activities of V184F (4.4-fold), F87Y (2.5-fold), I27F (2.1-fold) and V28Y (2.0-fold) towards *n*-dodecanal were greatly enhanced. G31F, A121F and M193Y exhibited comparable activity to WT towards *n*-dodecanal, and Y21R and I24Y displayed low activities towards *n*-dodecanal and *n*-decanal. Different behavior against *n*-decanal for I27F and V28Y were observed: improved activity for I27F and reduced activity for V28Y. C70F exhibited comparable activities to WT towards *n*-dodecanal and *n*-decanal. L198F showed reduced activity against *n*-dodecanal, but improved activity for *n*-decanal (1.4-fold). The activities of A121F, V184F and M193Y for *n*-decanal were improved. F87Y showed enhanced activity against *n*-decanal (1.8-fold) and similar activity to WT towards *n*-octadecanal.

### Activities towards medium- to short-chain aldehydes

#### a. Apparent k_cat_ values of WT cADO and mutants towards n-heptanal

*n*-Heptanal has been successfully used as the substrate to determine the apparent *k*_*cat*_ values of cADOs, and was utilized to examine whether the kinetics of the mutants were influenced (Fig. 2) [12–14]. The apparent *k*_*cat*_ value of M193Y was improved by about three-fold in comparison with that of WT, and was the highest one among all mutants. Compared with WT, the apparent *k*_*cat*_ values of L198F, I24Y and A121F were increased by two-fold, and C70F, Y21R, F87Y, V184F and G31F exhibited the comparable apparent *k*_*cat*_ values to WT, demonstrating that these mutations had little influence on the activities towards *n*-heptanal. The activities of I27F, V28Y and A118F were severely impaired.

#### b. Kinetic characterization of WT cADO and some mutants towards C_6–9_ aldehydes

**Fig. 3:**
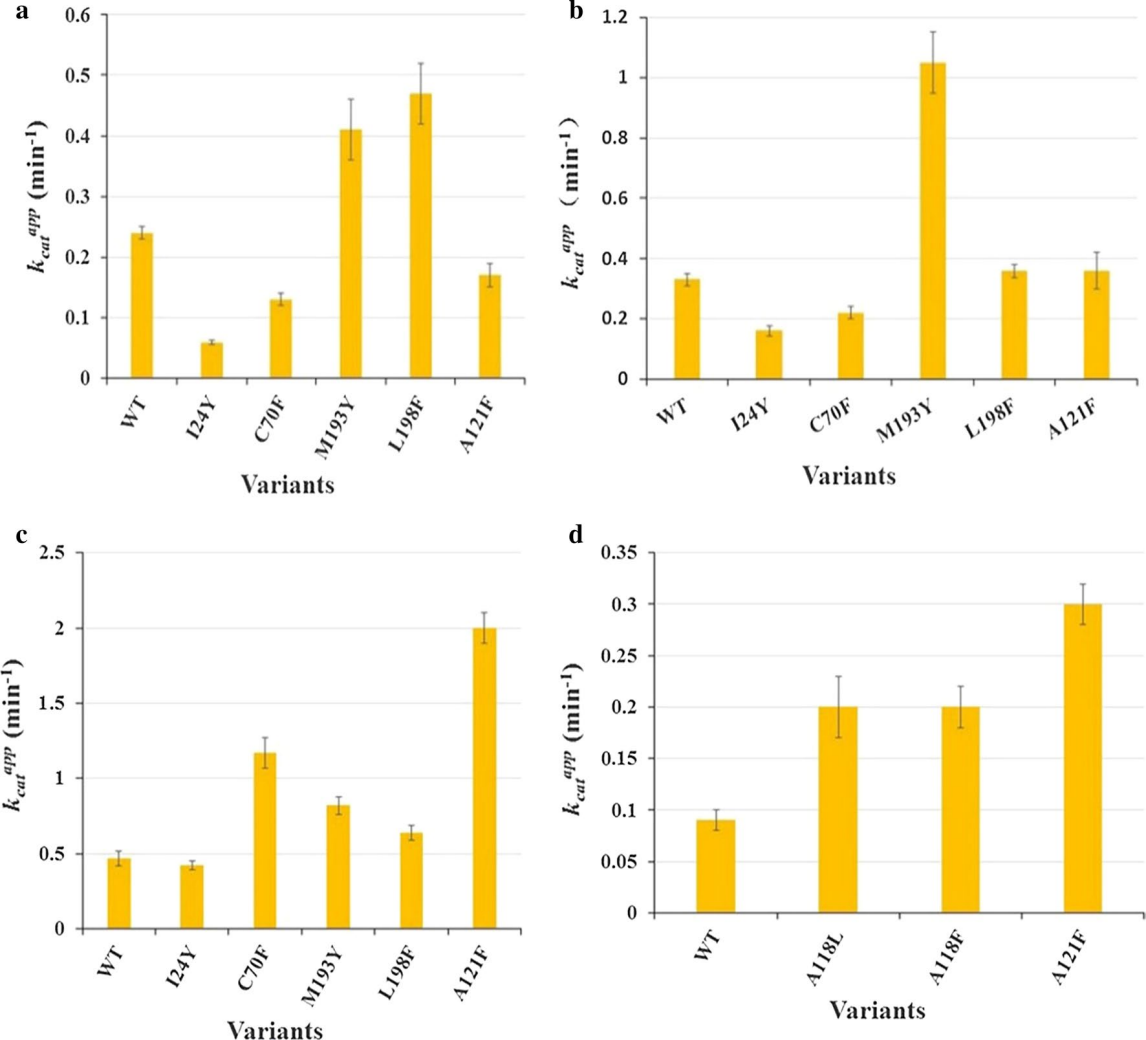
Apparent *k*_*cat*_ values of WT and variants towards *n*-nonanal (**a**), *n*-octanal (**b**), *n*-hexanal (**c**), and *n*-butanal (**d**). The amounts of *n*-octane, *n*-heptane, *n*-pentane, and *n*-propane produced were quantified by GC and a standard curve of known concentrations of the same product

C_6,8,9_ aldehydes were also used as the substrates to investigate the effects of some mutations on chain-length selectivity (Fig. 3). In comparison with WT, A121F, C70F, M193Y and L198F showed 2.7, 2.5, 1.7 and 1.4–fold increase in *k*_*cat*_^*app*^ against *n*-hexanal, respectively, and I24Y demonstrated similar *k*_*cat*_^*app*^ for *n*-hexanal. When *n*-octanal was used as the substrate, M193Y (3.2-fold) showed significantly improved activity, and A121F and L198F exhibited comparable activity to WT, and I24Y and C70F displayed much lower activity (Fig. 3b). While *n*-nonanal was used as the substrate, M193Y and L198F exhibited 1.7 and 2.0-fold increase in *k*_*cat*_^*app*^ respectively, and the apparent *k*_*cat*_ value of I24Y was much lower than that of WT, and those of C70F and A121F were about half of that of WT.

According to the published results by Khara et al. for cADO-PMT1231 from *Prochlorococcus marinus* (strain MIT9313), A134F, which is equivalent to A121F of cADO-1393, showed significantly improved activity against C_6,7,8_ aldehydes [21]. Therefore, the kinetic parameters of A121F against C_6–9_ aldehydes were determined in detail (Additional file 4). The kinetic parameters of A121F and WT cADO-1593 are listed in Table 1. In comparison with WT, A121F exhibited one-fold increase in the *K*_*m*_ value for *n*-nonanal and similar *K*_*m*_ value for other substrates. It seems that the *K*_*m*_ values of WT and A121F towards C_6–9_ aldehydes decrease with increasing chain-length of the substrates. This mutant displayed higher *k*_*cat*_ values for C_6,7_ aldehydes, but similar values against C_8,9_ aldehydes. The *k*_*cat*_ value of A121F towards *n*-hexanal was the highest among the substrates tested. Compared with WT, A121F showed significantly improved catalytic efficiency (*k*_*cat*_/*K*_*m*_) against *n*-hexanal and slightly higher one for *n*-heptanal. The catalytic efficiencies of WT towards C_8,9_ aldehydes are much higher than that of A121F for *n*-hexanal, which could be mainly caused by the big difference in *K*_*m*_ between them.

**Table 1:** Kinetic parameters of wild-type cADO and A121F towards C_6–9_ aldehydes

Aldehyde	*K*_*m*_ (mM)	*k*_*cat*_ (min^−1^)	*k*_*cat*_/*K*_*m*_ (mM^−1^min^−1^)
C_6_			
WT	0.93 ± 0.1	1.8 ± 0.1	1.9 ± 0.1
A121F	0.96 ± 0.05	6.9 ± 0.2	7.2 ± 0.3
C_7_			
WT	0.59 ± 0.07	0.79 ± 0.03	1.3 ± 0.1
A121F	0.56 ± 0.06	1.5 ± 0.1	2.7 ± 0.2
C_8_			
WT	0.18 ± 0.03	1.4 ± 0.06	7.8 ± 0.3
A121F	0.18 ± 0.036	0.98 ± 0.06	5.4 ± 0.3
C_9_			
WT	0.069 ± 0.0087	0.61 ± 0.02	9.0 ± 0.5
A121F	0.14 ± 0.022	0.77 ± 0.04	5.4 ± 0.3

#### c. Apparent k_cat_ values of WT cADOs and several mutants towards n-butanal

Since Gly31 and Ala118 are very close to the aldehyde group of the substrate, it is expected that G31F and A118F may exhibit inhibition against short aldehydes such as *n*-butanal than WT (Fig. 3d). In comparison with WT cADO-1593, the apparent *k*_*cat*_ value of A118F towards *n*-butanal was improved by 2.2-fold, whereas G31F showed greatly decreased activity for *n*-butanal (data not shown) (Fig. 3d). Considering that large Phe might have negative effects on activity, A118L was also constructed. Unexpectedly, A118L gave the same result as A118F). A121F exhibited the highest *k*_*cat*_^*app*^ value towards *n*-butanal (3.3-fold increase).

### Further characterization of WT and some cADO mutants

#### a. Circular dichroism (CD) for WT cADO and some mutants

**Fig. 4:**
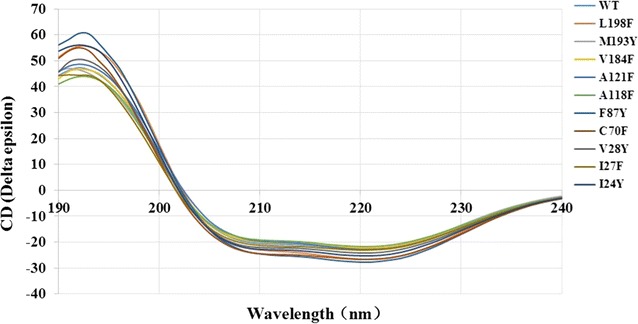
Circular dichroism (CD) spectra for WT cADO and some mutants. Far-UV CD spectra (190–260 nm) were recorded for protein samples (0.12 mg/mL) in 10 mM potassium phosphate buffer (pH 7.2) on a Jasco J-810 spectropolarimeter at 25 °C. Data were averaged over three runs and the background was subtracted

CD was used to investigate the effects of mutations on the conformational or structural changes. All mutants displayed similar CD spectroscopies to WT (Fig. 4), and secondary structures of WT cADO and all mutants were also very close (Additional file 5: Table S1). These results indicated that mutations did not lead to significant conformational changes for these mutants. Thus, the reasons why mutants showed different behavior (activities and/or chain length selectivity) from WT are due to the change of the side chains of amino acids instead of conformational or structural changes.

#### b. Mixed-substrate competition assays for WT and some cADO mutants

Based on the results of all mutants towards different chain-length substrates, it seems that mutants showed different chain length preference, for example, I24Y for *n*-heptanal, I27F for *n*-decanal and *n*-dodecanal, V28F for *n*-dodecanal, F87Y for *n*-decanal, C70F for *n*-hexanal, A118F for *n*-butanal, A121F for C_4,6,7_ aldehydes, V184F for *n*-dodecanal and *n*-decanal, M193Y for C_6–10_ aldehydes and L198F for C_7–10_ aldehydes. To further confirm the impact of mutations on chain length selectivity of cADO, the mixed-substrate competition assays were carried out for some mutants. While the preferred chain-length substrates of mutants were used, enzymatic activities of mutants were assayed in the presence of a short chain length substrate (*n*-butanal/*n*-heptanal/*n*-nonanal) and a long one (*n*-octadecanal), respectively.

When WT ADO was assayed against *n*-heptanal in the presence of the competition substrates *n*-butanal and *n*-octadecanal, respectively, both did not show obvious inhibition (Table 2). However, under same conditions, inhibition was observed for A121F towards *n*-heptanal in the presence of *n*-butanal and *n*-octadecanal. In contrast, *n*-butanal did not exhibit inhibition for I24Y against *n*-heptanal, whereas *n*-octadecanal displayed some inhibition. While *n*-octanal was used as the substrate, WT and M193Y demonstrated similar behavior to A121F in the presence of the competition substrates. WT and L198F performed differently in the presence of *n*-butanal using *n*-nonanal as the substrate: it did not inhibit WT, but inhibited L198F, whereas both could be inhibited by *n*-octadecanal.

**Table 2:** Apparent *k*_*cat*_ values of some cADO mutants and WT against the preferred substrates in the presence of the competition substrates

Mutants	Preferred/competition aldehydes	*k*_*cat*_^*app*^ (min^−1^)
WT	C_7_	0.80 ± 0.086
	C_7_/C_4_	0.80 ± 0.079
	C_7_/C_18_	0.77 ± 0.14
A121F	C_7_	1.41 ± 0.036
	C_7_/C_4_	0.75 ± 0.093
	C_7_/C_18_	0.69 ± 0.01
I24Y	C_7_	1.05 ± 0.076
	C_7_/C_4_	0.82 ± 0.024
	C_7_/C_18_	0.39 ± 0.026
WT	C_8_	0.33 ± 0.064
	C_8_/C_4_	0.35 ± 0.057
	C_8_/C_18_	0.24 ± 0.017
M193Y	C_8_	1.05 ± 0.20
	C_8_/C_4_	1.05 ± 0.18
	C_8_/C_18_	0.52 ± 0.032
WT	C_9_	0.24 ± 0.021
	C_9_/C_4_	0.23 ± 0.025
	C_9_/C_18_	0.18 ± 0.023
L198F	C_9_	0.50 ± 0.024
	C_9_/C_4_	0.25 ± 0.076
	C_9_/C_18_	0.25 ± 0.015

The apparent *k*_*cat*_ values of some cADO mutants and WT against the preferred substrates (2 mM C_7,8,9_ aldehydes) in the presence of the competition substrates (2 mM *n*-butanal or 150 μM *n*-octadecanal) were determined, respectively

When WT ADO, F87Y and I27F were assayed against *n*-dodecanal in the presence of *n*-heptanal and *n*-octadecanal, respectively, both exhibited inhibition for three enzymes to different extent (Table 3). In contrast, for V184F and V28Y, *n*-heptanal displayed some inhibition, whereas *n*-octadecanal did not.

**Table 3:** Yields of *n*-undecane of WT and some cADO mutants against *n*-dodecanal in the presence of the competition substrates

Mutants	Yield of *n*-undecane (μM)		
	C_12_	C_12_/C_7_	C_12_/C_18_
WT	1.95 ± 0.13	1.55 ± 0.12	1.61 ± 0.064
V184F	8.65 ± 0.0.28	4.04 ± 0.35	9.75 ± 0.038
F87Y	3.01 ± 0.0043	1.74 ± 0.16	1.50 ± 0.0091
I27F	4.30 ± 0.13	1.91 ± 0.061	2.27 ± 0.11
V28Y	3.51 ± 0.12	1.78 ± 0.31	3.19 ± 0.39

Yields of *n*-undecane of WT and some cADO mutants against *n*-dodecanal (150 μM) in the presence of the competition substrates (2 mM *n*-heptanal or 150 μM *n*-octadecanal) were determined by GC–MS

### Impact of engineered cADOs on distribution of fatty alkane profile in an *E. coli* cell factory

To demonstrate the importance of engineered ADO mutants on distribution of fatty alkane profile in vivo, V184F showing the highest activity for *n*-dodecanal was introduced into an engineered *E. coli* which can produce high titers of *n*-dodecanoic acid [28].

It is known that the fatty acid intermediate, such as fatty acyl-ACP or acyl–CoA, could be reduced by acyl-ACP or -CoA reductase into fatty aldehyde, which is further converted into fatty alk(a/e)ne by cADO [1]. Therefore, enhancing production of FFAs with certain chain-lengths is quite essential for fatty alk(a/e)ne production in genetically engineered *E. coli* [32]. To achieve this, the following strategies were used: (1) The gene *fadE* was knocked out, which can accumulate fatty acyl-CoA by blocking the fatty acid degradation pathway. (2) The gene *fadD* (an acyl-CoA synthetase) from *E. coli* was overexpressed to further boost fatty acyl-CoA yield. (3) The gene *BTE* encoding *Cinnamomum camphora* acyl-ACP thioesterase B was overexpressed to increase the abundance of medium-chain free fatty acids such as dodecanoic acid. (4) The gene *acr1* encoding fatty acyl-CoA reductase (FAR) from *Acinetobacter* sp. M-1 was overexpressed to reduce the accumulated fatty acyl-CoA into the corresponding fatty aldehyde. (5) Wild-type cADO or the mutant V184F was overexpressed to produce fatty alk(a/e)nes. The effects of overexpression of wild-type cADO and V184F on fatty alk(a/e)ne production in recombinant strain of *E. coli* BL21 (DE3) (Δ*fadE*) carrying *BTE* and *fadD* were investigated.

For the control strain, BL21 (Δ*fadE*), *n*-palmitic acid (C_16:0_) and *n*-steric acid (C_18:0_) were major FFAs (95 %) produced, together with trace quantities of *n*-dodecanoic acid (C_12:0_) and *n*-tetradecanoic acid (C_14:0_) (Table 4). The titers of *n*-dodecanoic acid of the recombinant strain of LB99 (co-expression of *BTE* and *fadD*) were improved by 5.5-fold compared to the control strain, while those of *n*-tetradecanoic acid, *n*-palmitic acid and steric acid were significantly reduced by 0.43-fold, 7.5-fold, and 23-fold, respectively (Table 5). A large quantity of *n*-dodecanol was also detected in LB99, which could be caused by some endogenous reductases in *E. coli* [33]. The engineered strains LB100 (co-expression of *BTE*, *fadD,* and wild-type *cADO* gene) and LB101 (co-expression of *BTE*, *fadD,* and cADO mutant V184F gene) produced similar titers of *n*-dodecanoic acid, which were higher than that of the control strain and lower than that of LB99. These results indicated that co-overexpression of *fad* and *BTE* dramatically improved the titers of *n*-dodecanoic acid in BL21 (Δ*fadE*).

**Table 4:** Fatty alk(a/e)ne production in genetically engineered *E. coli* strains

mg/L/OD	ΔfadE (BL21)	LB99	LB100	LB101
*n*-Undecane	ND	ND	0.044 ± 0.0030	0.078 ± 0.0010
*n*-Pentadecane	ND	ND	0.066 ± 0.011	0.016 ± 0.0028
8-Heptadecene	ND	ND	0.30 ± 0.023	ND
*n*-Dodecanol	ND	0.72 ± 0.0035	ND	ND
*n*-Dodecanoic acid	0.16 ± 0.014	1.1 ± 0.12	0.41 ± 0.039	0.50 ± 0.060
*n*-Tetradecanoic acid	1.43 ± 0.050	1.0 ± 0.015	1.0 ± 0.16	0.95 ± 0.055
*n*-Hexadecanoic acid	15.1 ± 2.1	1.8 ± 0.030	2.8 ± 0.32	4.1 ± 0.27
*n*-Octadecanoic acid	14.9 ± 1.6	0.62 ± 0.025	1.1 ± 0.12	0.67 ± 0.077

Yields of fatty alk(a/e)nes and others produced by genetically engineered *E. coli* strains were quantified by GC–MS

*ND* not determined due to low concentrations

**Table 5:** Detailed information on the plasmids and strains used

Plasmids or strains	Relevant characteristics	Reference
pET-28a	Kan^r^; pBR322 ori; lacI; T7 promoter	Novagen
pAL134	Kan^r^; pET-28b derivative containing *acr1* gene; T7 promoter	[34]
pKC11	Ap^r^; pFN476 derivative containing *E. coli fadD*; BAD promoter	[34]
pLB1593-acr1	Kan^r^; pET-28a derivative containing *acr1* and wild-type cADO gene; T7 promoter	This study
pLB1593-V184F-acr1	Kan^r^; pET-28a derivative containing *acr1* and cADO mutant V184F gene; T7 promoter	This study
BL21 (ΔfadE)	BL21 (DE3) knocking out *fadE*	[33]
LB99	BL21 (Δ*fadE*) bearing pAL134 and pKC11	This study
LB100	BL21 (Δ*fadE*) bearing pAL134, pKC11 and pLB1593-acrI	This study
LB101	BL21 (Δ*fadE*) bearing pAL134, pKC11 and pLB1593-V184F-acrI	This study

No fatty alk(a/e)nes were detected in BL21 (Δ*fadE*) and engineered strain LB99, but about 0.72 mg/L/OD *n*-dodecanol was detected in LB99 cultures. LB100 produced 0.044 mg/L/OD *n*-undecane, 0.066 mg/L/OD *n*-pentadecane and 0.3 mg/L/OD 8-heptadecene. Compared to LB100, LB101 produced improved titers of *n*-undecane (1.7-fold) and significantly reduced titers of *n*-pentadecane (0.24-fold). In addition, no 8-heptadecene was detected in LB101 cultures (Additional file 6: Figure S3).

## Discussion

The carbon number distribution of fatty alk(a/e)nes varies in different fuels such as gasoline, diesel and jet fuel, and has important effects on the properties of fuels. Therefore, it’s significant to genetically control the carbon chain-length of microbial hydrocarbons [27]. The available crystal structures of cADOs with fatty acids or fatty alcohol or substrate analog bound have enabled structure-guided substrate specificity engineering of cADO [1, 17, 21, 23]. In this current paper, we have identified some potential amino acids which might impact the substrate chain-length specificity of cADO through structural analysis of cADOs. All selected amino acids are adjacent to the aldehyde group and the hydrophobic tail of the substrate and along the substrate pocket. We hypothesized that the chain-length selectivity of cADO might be changed or at least substrate access would be influenced when these amino acids were replaced with the large ones.

All mutants except F87Y showed greatly reduced or no activity (A118F) towards long-chain aldehydes (C_14,16,18_), and demonstrated preference against <C_14_ aldehydes, supporting our hypothesis that replacement of the amino acids with the large ones resulted in hindering access of long-chain substrates (≥C_14_) (Figs. 2, 3). The results are consistent with the relative positions of these amino acids in the crystal structure along the substrate channel (Fig. 1). Therefore, the size of the hydrophobic channel of the substrate was successfully decreased. Most of these amino acids could be useful for engineering cADO to synthesize fatty alk(a/e)nes (<C_13_).

Gly31 and Ala118 are close to the aldehyde group of the substrate. According to the predicted orientation of the side chains of G31F and A118F by PyMOL (Additional file 7: Figure S4), both mutants present their side chains approximately towards the C_4_ position of the bound ligand, consistent with their performance against C_14,16,18_ aldehydes. However, they performed differently towards other substrates such as C_4,7,12_ aldehydes. The results are not in agreement with the expected orientation of the side chains of G31F and A118F (Additional file 7: Figure S4). A118F had significant effects on substrate specificity, whereas G31F did not. Considering the relative position and the different performance of G31F and A118F, it appears that amino acid 31 is in a more flexible position than amino acid 118. To achieve propane production by cyanobacteria, Ala118 is a good candidate for further protein engineering.

In the case of the mutants of the amino acids along the substrate channel, including I24Y, I27F, V28Y, F87Y, A121F, V184F, M193Y and L198F, they behaved differently, and were discussed according to their relative positions to the bound substrate analog: (1) Phe87 protrudes the side chain to the C_5_ position. Given the small difference between the side chains of Phe and Tyr, it is understandable that introducing an additional hydroxyl group might not have big impact on substrate specificity and activity, except for *n*-dodecanal (2.5-fold improvement). (2) Ile27 and Val28 are in the relatively similar position, presenting their side chains towards the C_7_–C_8_ position of the substrate analog. However, the results demonstrated that mutation of them into the large ones caused some steric hindrance for long-chain substrates (≥C_14_), and the activities of I27F and V28Y against *n*-dodecanal are about twofold higher than that of WT. Khara et al. reported the similar results for V41Y (the counterpart of V28Y of cADO-1593) and WT of cADO-PMT1231 against the substrates with different chain-lengths [21]. (3) Though the side chains of both Val184 and Leu198 points towards the C_9_–C_10_ position, V184F and L198F performed differently. Mutation of Leu198 into large Phe might have negative impact on substrate binding (>C_9_ aldehydes). The results are consistent with the expectation: L198F exhibited dramatically reduced activity towards *n*-dodecanal and higher chain-length selectivity against aldehydes (≤C_10_), especially for *n*-heptanal. By contrast, substitution of Val184 by large Phe resulted in hindered access of long chain-length substrates (≥C_14_). V184F showed significantly improved activity towards *n*-dodecanal (the highest among all mutants) and similar activity to WT for *n*-heptanal. Though both Leu198 and Val184 present the side chains towards the similar position of the substrate, V184F and L198F demonstrated different behavior (chain length preference), especially towards *n*-dodecanal. (4) Mutagenesis of Ala121 into Phe blocked access of long-chain length aldehydes (>C_12_). The side chain of Ala121 points approximately towards the C_10_–C_11_ position of the substrate analog, which is consistent with the substrate selectivity of A121F. The results of A121F against the tested substrates are very similar to those of A134F of PMT1231 (corresponding to A121F of cADO-1593), and further proved the significance of this amino acid for improving the chain-length selectivity of cADO. A121F showed preference towards ≤C_12_ aldehydes, higher preference for C_4,6,7_ aldehydes and highest for *n*-hexanal (Figs. 2, 3; Table 1). The results suggested that A121F exerted great effects on both substrate preference and activity. (5) Ile24 presents the side chain towards the C_12_–C_13_ position, and the results indicated that mutation of Ile24 into large Tyr affected access of medium to long-chain length substrates (≥C_9_). I24Y showed higher preference for *n*-heptanal. (6) The side chain of Met193 points to the C_13_–C_14_ position, thus mutation of Met193 into large Tyr could lead to hinder binding of aldehydes (>C_12_) to enzymes. M193Y showed the highest activity against *n*-heptanal. Finally, it is worth pointing out that the inconsistency between predicted and actual chain-length selectivity was observed for some mutants such as I27F, V28Y, V184F, I24Y and M193Y.

Tyr21 and Cys70 are close to the hydrophobic tail of the substrate. Mutation of Tyr21 into long and hydrophilic Arg impeded access to long chain-length aldehydes (≥C_12_), whereas replacement of Cys70 with large Phe hindered access of long chain-length aldehydes (>C_12_) (Figs. 2, 3). Y21R did not show any preference towards tested substrates, whereas C70F displayed highest activity for *n*-hexanal. It has been reported that C71A/S (Cys71, equivalent to Cys70 of cADO-1593) of cADO from *Nostoc punctiforme* PCC 73102 reduced the hydrocarbon producing activity of cADO and facilitated the formation of a dimer [22]. Based on the results and the positions of Tyr21 and Cys70 in the crystal structure, we predicted that both amino acids are possibly involved in the substrate entrance. However, according to the crystal structure of the complex of PMT1231 with the substrate (11-(2-(2-Ethoxyethoxy)ethoxy)undecanal) (PDB code: 4PGI, L194A of PMT1231 complexed with the substrate), Marsh et al, observed a T-shaped region of electron density for the bound substrate, and suggested that the fork of exiting close to Leu194 of PMT1231 might be the substrate entry point (Additional file 8: Figure S5) [23]. Our results seem not to support this. Meanwhile, Marsh et al, found that L194A of PMT1231 had similar kinetic properties to WT implying that L194A does not play a key role in limiting substrate access to the active site. Thus, the fork of the T-shaped region for the complexed substrate occupying the cavity to bind fatty acids is the possible substrate entry point.

Substrate competition experiments reflected the different binding affinities between the preferred and competition substrates (Tables 2, 3). It seems that WT binds to *n*-octadecanal more tightly than to *n*-butanal and WT showed different binding affinities towards C_7,8,9_ aldehydes, which are consistent with the corresponding *K*_*m*_ values of WT against them (Tables 1, 2, 3). The results of A121F and L198F suggested that both mutants showed improved binding affinities towards *n*-octadecanal and *n*-butanal. In comparison, the competition results of I24Y and M193Y indicated that enhanced binding affinities towards *n*-octadecanal were observed for them and those against *n*-butanal did not change a lot. While *n*-dodecanal was used as the preferred substrate in the presence of the competition substrates *n*-octadecanal and *n*-heptanal, similar conclusions were drawn. Enhanced binding affinities against *n*-octadecanal and *n*-heptanal for F87Y and I27F were observed. V184F and V28Y showed increased binding affinities towards *n*-heptanal, and the binding affinities of V184F and V28Y for *n*-octadecanal were not changed.

As discussed above, all mutants except F87Y exhibited lower activities against C_18,16,14_ aldehydes than WT (Fig. 2a, b), whereas V184F showed the highest activity for *n*-dodecanal among all mutants and comparable activity to WT towards *n*-heptanal (Fig. 2c, d). It seems that the replacement of Val184 with Phe had important effects on substrate binding with chain-length preference, but no big influence on enzymatic activity. Therefore, V184F was chosen for further investigation. It was introduced into engineered *E. coli* producing high titer of *n*-dodecanoic acid to see if the carbon chain-length selectivity of cADO mutants could be used to control the carbon chain-length distribution of fatty alk(a/e)nes in vivo [28]. The results of fatty alk(a/e)ne production in genetically engineered *E. coli* suggested that cADO was successfully engineered for *n*-undecane production in *E. coli* and introduction of the mutant V184F had significant influence on distribution of fatty alk(a/e)ne profile in *E. coli* (Table 4). Thus, V184F could be potentially used for *n*-undecane production by genetically engineered microbial cell factories in future.

## Conclusions

Some amino acids, which could affect the substrate specificity of cADO were identified based on the crystal structure of cADO with the bound substrate analogs and kinetically characterized. The substrate preferences of some mutants towards different chain-length substrates were successfully enhanced through structure-orientated protein engineering. The in vivo experiments of V184F in genetically engineered *E. coli* demonstrated the impact of structure-guided engineering of cADOs on the distribution of the fatty alk(a/e)ne profile. The study would deepen our understanding of the structure–function relationship of cADOs, and provide a guide for designing cADO to produce fatty alk(a/e)nes with certain chain lengths.

## Methods

### Materials

Oligonucleotide synthesis and DNA sequencing were carried out by Sangon Biotech Co. Ltd (Shanghai, China) or Sunny (Shanghai, China). The codon-optimized *Synpcc7942_1593* encoding ADO from *Synechococcus elongatus* PCC7942 was synthesized by Sangon Biotech Co. Ltd). *Pfu* DNA polymerases and *Dpn*I were from Fermentas (Pittsburgh, Pennsylvania, USA). The kits used for molecular cloning were purchased from Omega (USA) or Takara (Japan). *n*-Butanal, C_6–10_ aldehydes, *n*-dodecanal, C_14,16,18_ alcohols, Dess-Martin reagent, *n*-pentadecanol, *n*-eicosane, *n*-heptadecanoic acid, BSA (Bovine Serum Albumin), NADH, catalase, DMSO, phenazine methosulfate (PMS), NTA (Nitrilotriacetic acid) and ferrous ammonium sulfate were obtained from Sigma-Aldrich. Nickel column was from Novagen. Amicon YM10 membrane was from Millipore.

### Bacterial strains, plasmids and media

*E.coli* DH5α and BL21(DE3) were, respectively used for routine DNA cloning and protein expression. *E. coli* strains were grown in LB broth or terrific broth media containing antibiotics at standard concentrations. 50 μg/mL Kanamycin was added when required.

*E. coli* BL21 (Δ*fadE*) was used as host cells for fatty alk(a/e)ne production [34]. Plasmid pAL143 expressing acyl-ACP thioesterase BTE from *Cinnamomum camphora* with P_BAD_ promoter was utilized to overproduce free fatty acid (C12:0) in the host cells [35]. Plasmid pKC11 encoding *E. coli fadD* gene with pSC101 origin was employed to overexpress an acyl-CoA synthase and block β-oxidation pathway [35]. To co-express *acr1*, a fatty acyl-CoA reductase and pET-28a-1593 (or pET-28a-1593-V184F), the *Bgl* II-*Spe* I double-digested DNA fragment of pAL134 was inserted into pET-28a-1593 cut by *Bgl* II and *Xba* I, resulting in pLB1593-acr1 (or pLB1593-V184F-acr1) [35]. Engineered *E. coli* strains were constructed by transformation BL21 (Δ*fadE*) with the plasmids in Table 5.

### Construction of site-directed mutants

General molecular biology techniques were carried out by standard procedures [36]. Plasmid DNA was isolated using the Plasmid Mini Kit I. Site-directed mutants were constructed according to the standard QuikChange Site-Directed Mutagenesis protocol (Stratagene Ltd, La Jolla, California, USA) using pET28a-1593 as a template and the primers listed in Table S1 (Additional file 9: Table S2). The required mutations were confirmed by DNA sequencing.

For construction of double/triple/multiple mutants, pET28a-1593 harboring single or double or triple mutation(s) was used as a template following the same protocol as above.

### Protein overexpression and purification

Wild-type cADO 1593 and the mutants were overexpressed in *E. coli* BL21(DE3) following the published procedure [13]. The plasmids were transformed into *E. coli* BL21(DE3) competent cells. Terrific broth media at 37 °C was utilized for protein expression. The cultures were induced with 1 mM IPTG supplemented with 50 μM ferrous ammonium sulfate and 50 μg/mL kanamycin when OD_600nm_ reached around 0.6. The cells were continuously grown for additional 3.5 hours before being harvested at 37 °C, 220 rpm. The cultures were then disrupted by sonication in binding buffer The recombinant protein was washed using binding buffers containing a gradient (30 to 250 mM imidazole) at 4 °C. SDS-PAGE was performed in 12 % polyacrylamide gel using Coomassie Blue R-250 staining. The buffer containing 1 mM EDTA and 1 mM NTA was utilized to dialyze the protein for preparing apo-cADO-1593, and stoichiometric amounts of ferrous ammonium sulfate was added to reconstitute the diferrous form of cADO-1593 prior to assay. Proteins were concentrated and the concentration was determined by the Bradford method using bovine serum albumin as a standard [36].

### Synthesis of C_14,16,18_ aldehydes

According to the published procedure, C_14,16,18_ aldehydes were synthesized, respectively using the corresponding fatty alcohols as the starting materials [11, 37]. The synthesized products were confirmed by GC–MS.

### Enzyme assay

According to the published procedure [13], assays were carried out in HEPES buffer, containing 100 mM KCl and 100 mM HEPES, pH 7.2 (Additional files 4, 10). The reaction mixtures contain NADH (750 μM), catalase (1 mg/mL), ferrous ammonium sulfate (80 μM), PMS (75 μM), appropriate amount of aldehydes (150 μM for C_12,14,16,18_ aldehydes, 2 mM for C_4,6,7,9,10_ aldehydes), cADO (10 μM for *n*-decanal, 20 μM for ≥C_12_ aldehydes, 2 μM for ≤C_9_ aldehydes). *n*-Eicosane (10 μM) was used as an internal standard for nonvolatile C_11,13,15,17_ alkanes. Ethyl acetate (500 μL) was then added to terminate and to extract the reactions for C_10,12,14,16,18_ aldehydes after being vibrated by Vortex-Genie 2 for 1 hour at 37 °C. A 400 μL extractant was then analysed by GC–MS. All kinetic assays were carried out in triplicate.

### Quantitation of nonvolatile C_11,13,15,17_ alkanes by gas chromatography-mass spectrometry (GC–MS)

Quantification of nonvolatile C_11,13,15,17_ alkanes was performed by gas chromatography-mass spectrometry (GC–MS). GC–MS analysis was performed on an Agilent 7890A gas chromatograph equipped with a split/split less capillary inlet, an Agilent 5975C GC/MSD with Triple-Axis Detector and an Agilent 7683B automatic liquid sampler (ALS). A HP-WAX column (30 m × 0.25 mm × 0.25 µm) was utilized with the following oven temperature program: 40 °C held for 5 minutes, to 240 °C at 25 °C min^−1^, and held for 15 minutes. The injector temperature was 250 °C (split less injection), and the carrier gas employed was helium at a flow rate of 1 mL min^−1^.

### Gas chromatography detection of volatile C_3,5,6,7,8_ alkanes

The C_3,5,6,7,8_ alkane products were quantified by detecting headspace of the reactions using gas chromatography (GC). At time intervals (0, 1, 2.5, 5, 7.5 min for >C_4_ aldehydes and 0, 1, 2.5, 3.5, 5 min for *n*-butanal), the reactions were terminated by being laid on ice. The mixtures were shaken at 37 °C and 200 rpm unless specified otherwise. Reactions of propane and pentane were performed at room temperature without being shaken due to their low boiling point. All assays were performed in triplicate.

Detection and quantification of the alkane products were performed on a Varian 3800 GC equipped with a HP-INNOWAX column (30 m × 0.25 mm × 0.25 μm). The column temperature was programmed as follows: 63 °C held for 6 minutes (for detection of propane and pentane) and 63 °C held for 1 minute, to 120 °C at 20 °C min^−1^ (for detection of *n*-hexane, *n*-heptane and *n*-octane). FID temperature was set at 200 °C and the injector temperature was 200 °C (20:1 split). The carrier gas helium was at a flow rate of 1 mL min^−1^. Pure alkane standards were utilized to identify and quantitation of each alkane.

### Fatty alk(a/e)ne production and analysis in genetically engineered *E. coli* strains

A single colony was cultured in LB medium overnight and then inoculated into modified mineral medium at 30 °C [1]. Cells were grown in the presence of kanamycin (25 mg/mL for pLB1593-acr1 and pLB1593-V184F-acr1), ampicillin (50 mg/mL for pKC11) and chloramphenicol (17 mg/mL for pAL143). P_BAD_ promoter and P_T7_ were, respectively induced with 0.4 % l-arabinose and 0.5 mM isopropyl β-d-thiogalactoside at an OD_600nm_ of 0.6–0.8. Cell cultures were induced for 24 hours.

Cell cultures were then mixed thoroughly with equivalent volume of chloroform–methanol (v/v, 2:1), together with *n*-pentadecanol, *n*-eicosane and *n*-heptadecanoic acid as internal standards [34]. As described earlier, cells were prepared and analyzed for alk(a/e)ne production [34]. The temperature of the injector was set at 250 °C and the column temperature was programmed as follows:100 °C for 1 minute, then increase of 5 °C/min to 200 °C and increase of 25 °C/min to 240 °C and held for 15 minutes.

### Circular dichroism spectroscopy

Far-UV CD spectra (190 to 260 nm) were measured for protein samples (0.12 mg/mL) in 10 mM potassium phosphate buffer (pH 7.2) on a Jasco J-810 spectropolarimeter at 25 °C. Data were averaged over three runs and the background was subtracted.

Secondary-structure analyses were performed with BeStSel method [38], which is available at the bestsel.elte.hu server.

### Substrate competition assays for some engineered proteins and WT

In the substrate competition assays, the preferred substrates for the mutants were added together with a shorter- or longer-chain one as the competition substrate.

For mutants A121F and I24Y, *n*-heptanal (2 mM) was added together with equal molar *n*-butanal or *n*-octadecanal (150 μM). The apparent *k*_*cat*_ values of the engineered proteins for *n*-heptanal were determined as above. *n*-Octanal and *n*-nonanal were, respectively employed to evaluate the substrate preference for M193Y and L198F in the same way. In the assays for mutants V184F, F87Y, I27F and V28Y, *n*-dodecanal was used together with *n*-heptanal (2 mM) or *n*-octadecanal (150 μM). *n*-Decane was employed as an internal standard to evaluate the production of *n*-undecane. The composition of the reaction mixture and reaction time were same as those in enzyme assays.

## Additional files

10.1186/s13068-016-0596-9 Structural superimposition of 1593 in cyan (4RC5) and PMT1231 in green (4KVQ) with the substrate analogs bound and shown.
10.1186/s13068-016-0596-9 SDS PAGE for WT and all cADO variants.
10.1186/s13068-016-0596-9 Relative activity of WT and cADO mutants against different substrates.
10.1186/s13068-016-0596-9 Original data for determination of kinetic parameters of A121F and WT cADO against C6,7,8,9 aldehydes.
10.1186/s13068-016-0596-9 Secondary structure estimation based on CD spectra of WT and cADO variants.
10.1186/s13068-016-0596-9 Comparison of fatty alk(a/e)ne production in *E. coli* strains harboring WT cADO and V184F.
10.1186/s13068-016-0596-9 Predicted orientations of A118F and G31F by PyMOL.
10.1186/s13068-016-0596-9 Part of superimposed structures of 1593 (PDB code: 4RC5) and PMT1231 (PDB code: 4PGI)
10.1186/s13068-016-0596-9 Primers used for construction of site-directed mutants.
10.1186/s13068-016-0596-9 Original data for determination of apparent kcat values of WT and mutants.

## Abbreviations

ACP: acyl carrier protein
ADO: aldehyde-deformylating oxygenase
Fd: ferredoxin
FNR: ferredoxin-NADP^+^ reductase
PMS: phenazine methosulfate
GC-MS: gas chromatography–mass spectrometry
