# Supplementary material for: Structure-oriented substrate specificity engineering of aldehyde-deformylating oxygenase towards aldehydes carbon chain length
Source: Biotechnol Biofuels. 2016 Aug 31;9(1):185. doi: 10.1186/s13068-016-0596-9 (PMC5007808; doi:10.1186/s13068-016-0596-9)

**Additional file 1**

**Figure S1** Structural superimposition of 1593 in cyan (4RC5) and PMT1231 in green (4KVQ) with the substrate analogs bound and shown


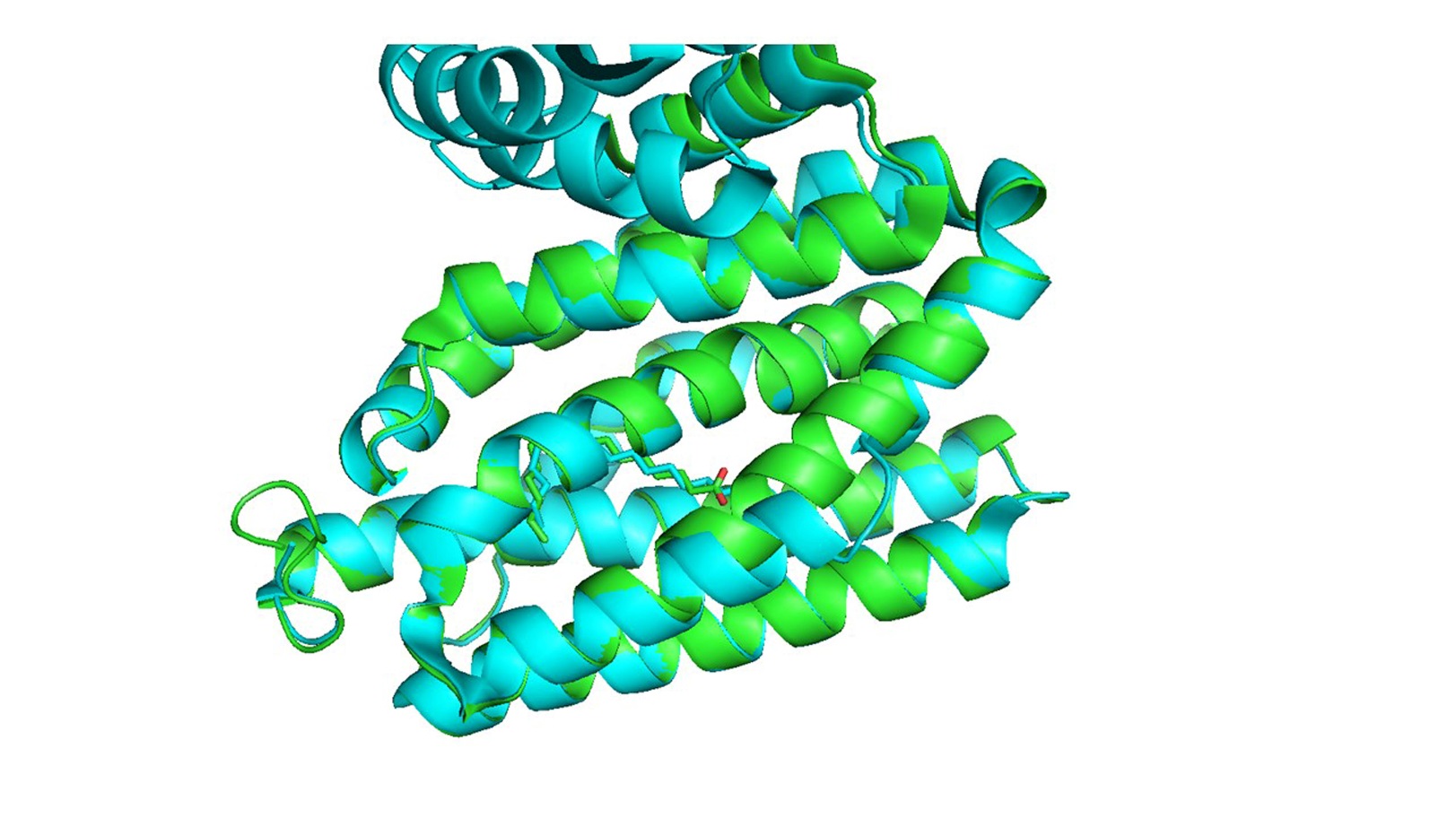

Supplement: Supplementary file 1 — 10.1186/s13068-016-0596-9 Structural superimposition of 1593 in cyan (4RC5) and PMT1231 in green (4KVQ) with the substrate analogs bound and shown. [file 13068_2016_596_MOESM1_ESM.docx]
