# Supplementary figures and images for: Structure-oriented substrate specificity engineering of aldehyde-deformylating oxygenase towards aldehydes carbon chain length
Source: Biotechnol Biofuels. 2016 Aug 31;9(1):185. doi: 10.1186/s13068-016-0596-9 (PMC5007808; doi:10.1186/s13068-016-0596-9)

**Additional file 2**

**Figure S2 SDS PAGE for WT and all cADO variants**

**
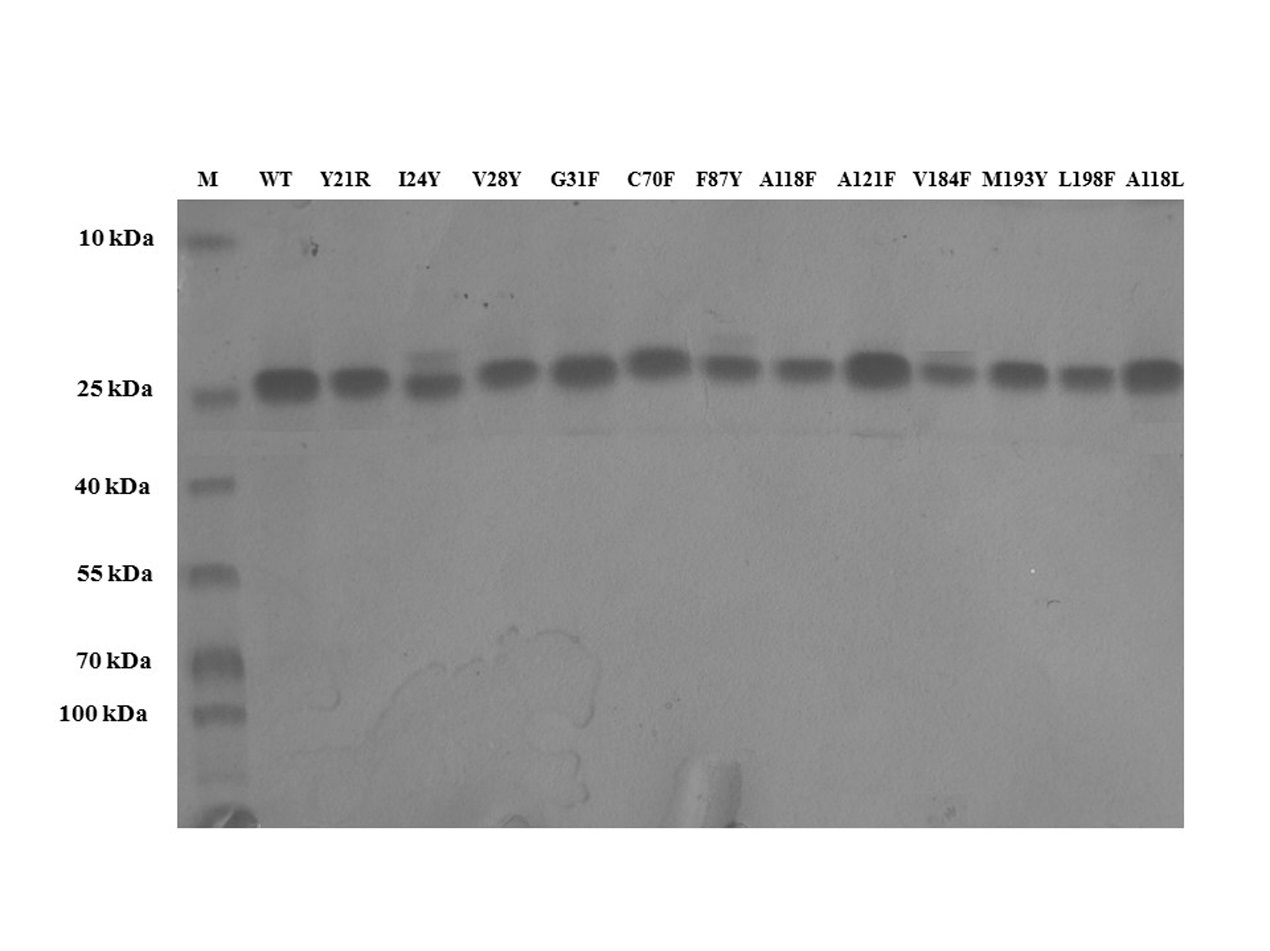
**

Supplement: Supplementary file 2 — 10.1186/s13068-016-0596-9 SDS PAGE for WT and all cADO variants. [file 13068_2016_596_MOESM2_ESM.docx]

**Additional file 7**

**Figure S4** Predicted orientations of A118F and G31F by PyMOL

A118F


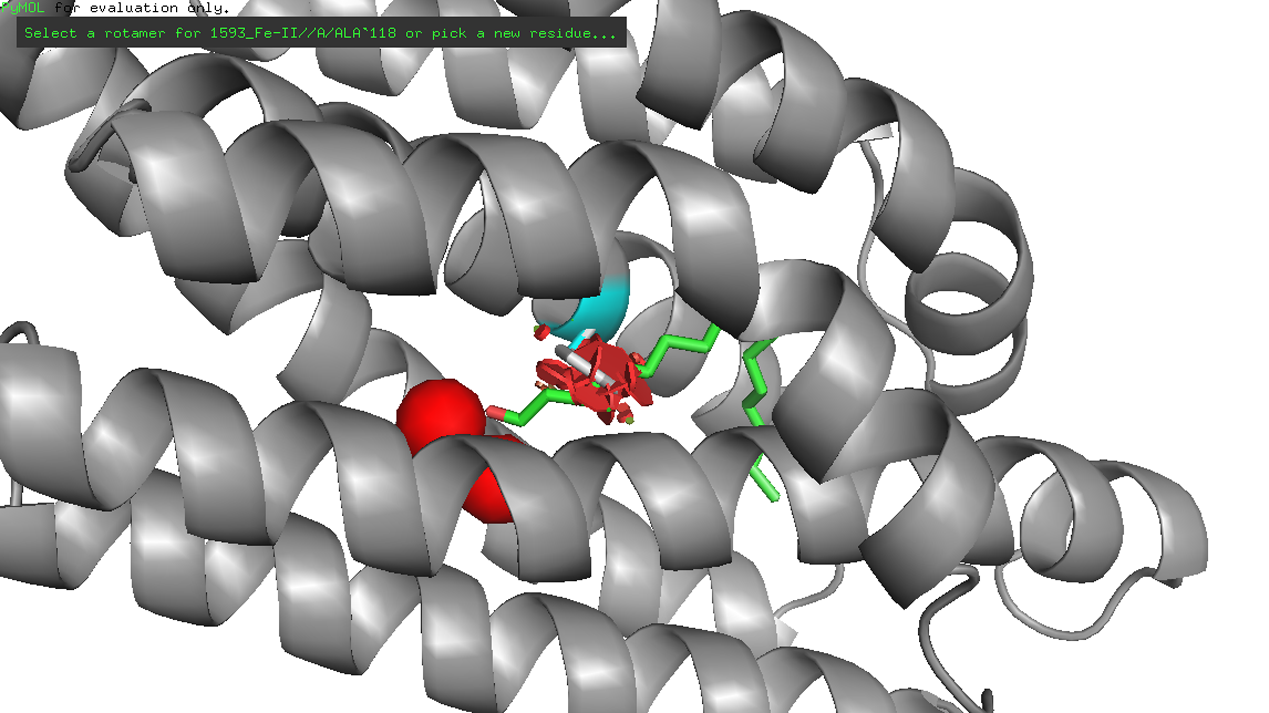


G31F


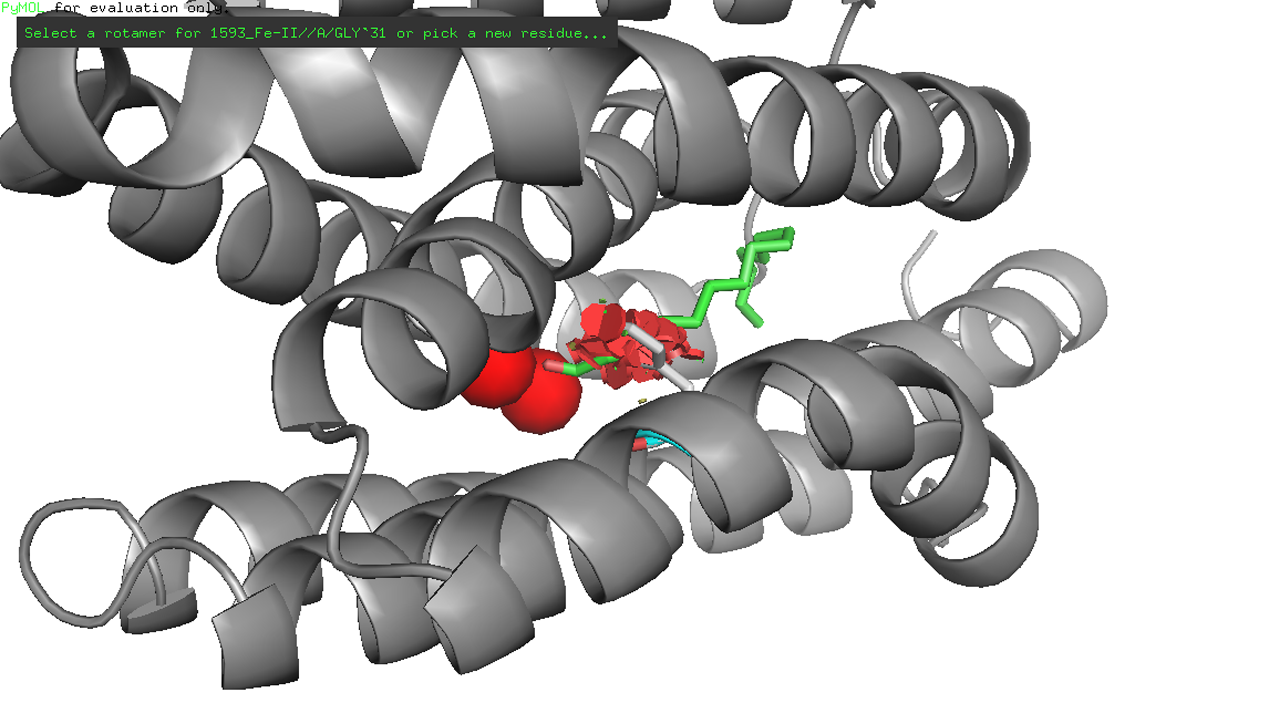

Supplement: Supplementary file 7 — 10.1186/s13068-016-0596-9 Predicted orientations of A118F and G31F by PyMOL. [file 13068_2016_596_MOESM7_ESM.docx]
