# Supplementary material for: Structure-oriented substrate specificity engineering of aldehyde-deformylating oxygenase towards aldehydes carbon chain length
Source: Biotechnol Biofuels. 2016 Aug 31;9(1):185. doi: 10.1186/s13068-016-0596-9 (PMC5007808; doi:10.1186/s13068-016-0596-9)

**Additional file 3**

**Relative activity of WT and cADO mutants against different substrates**

1. Relative activity of wild-type cADO and variants for C_16,18_ aldehydes


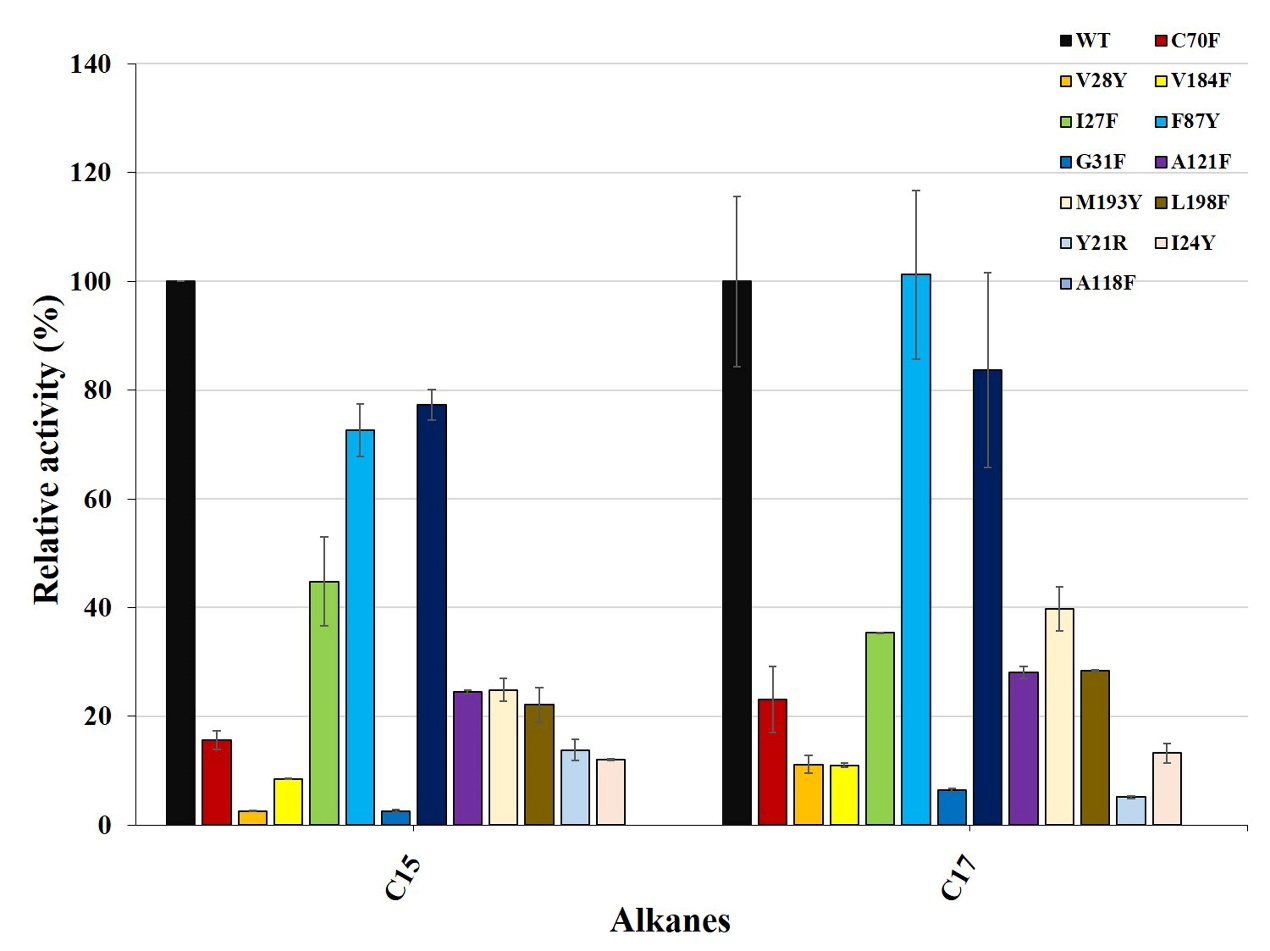


1. Relative activity of wild-type cADO and variants for C_12,14_ aldehydes


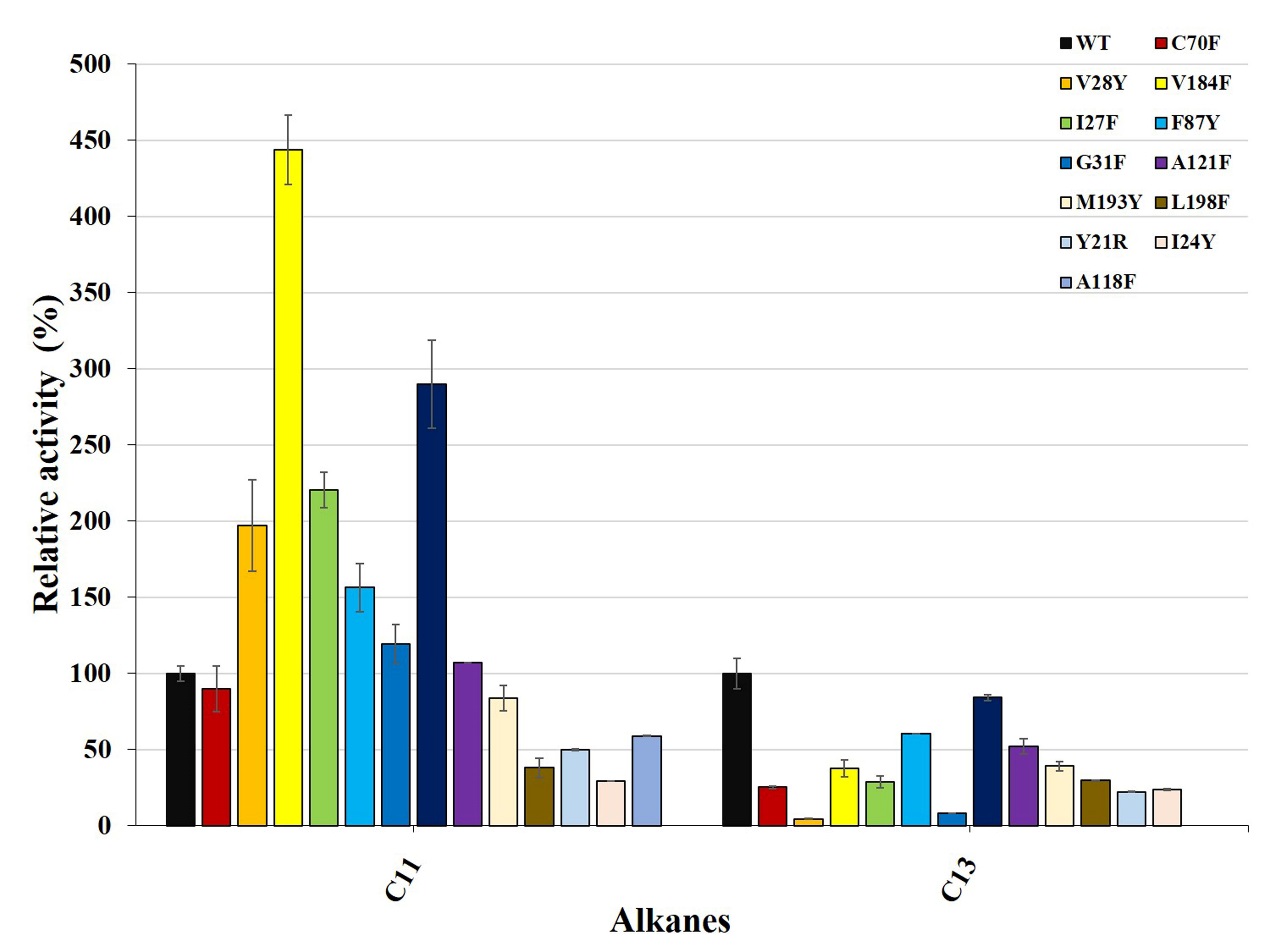


1. Relative activity of wild-type cADO and variants for *n*-decanal


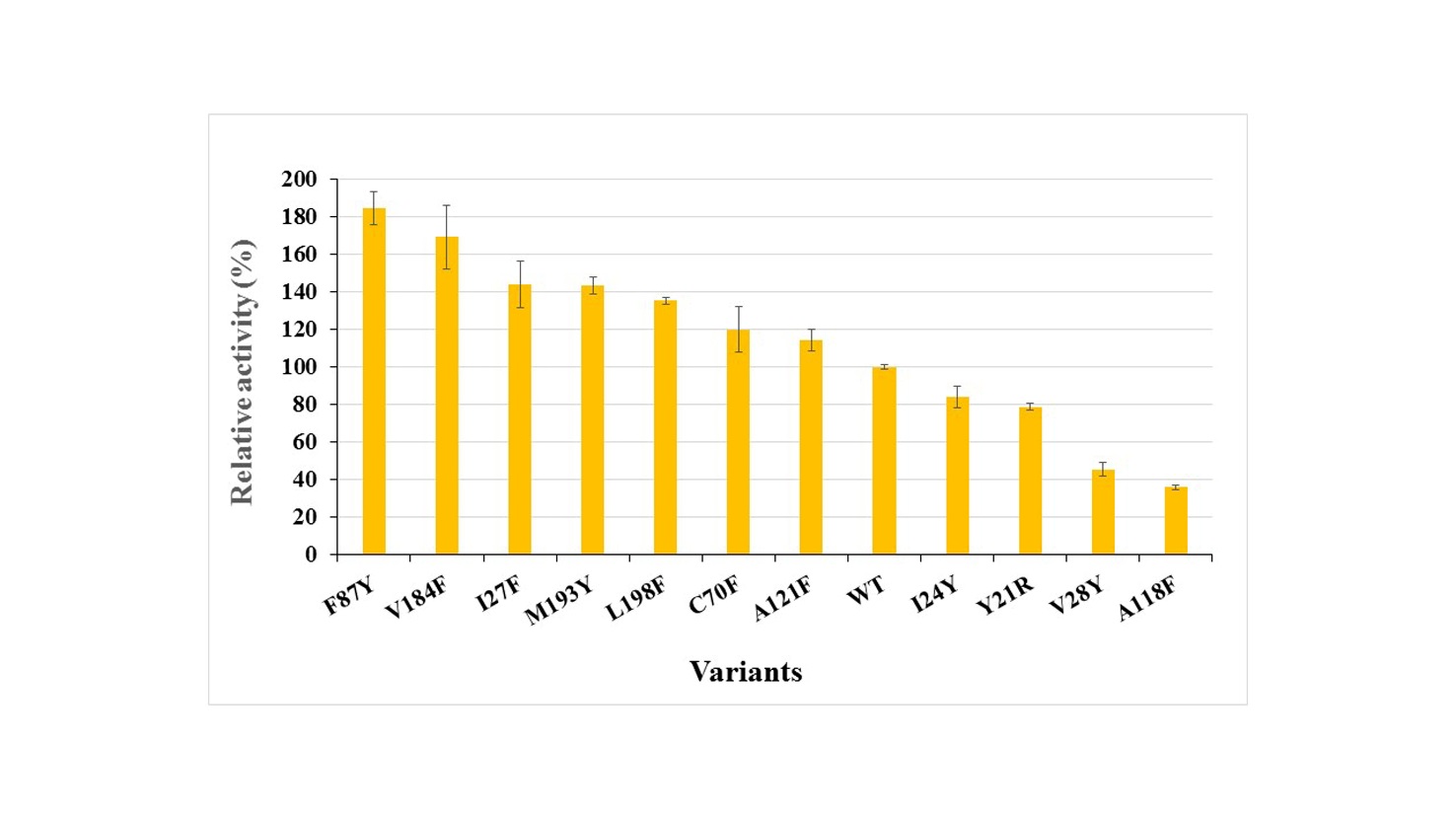


1. Relative activity of WT and variants towards *n*-heptanal


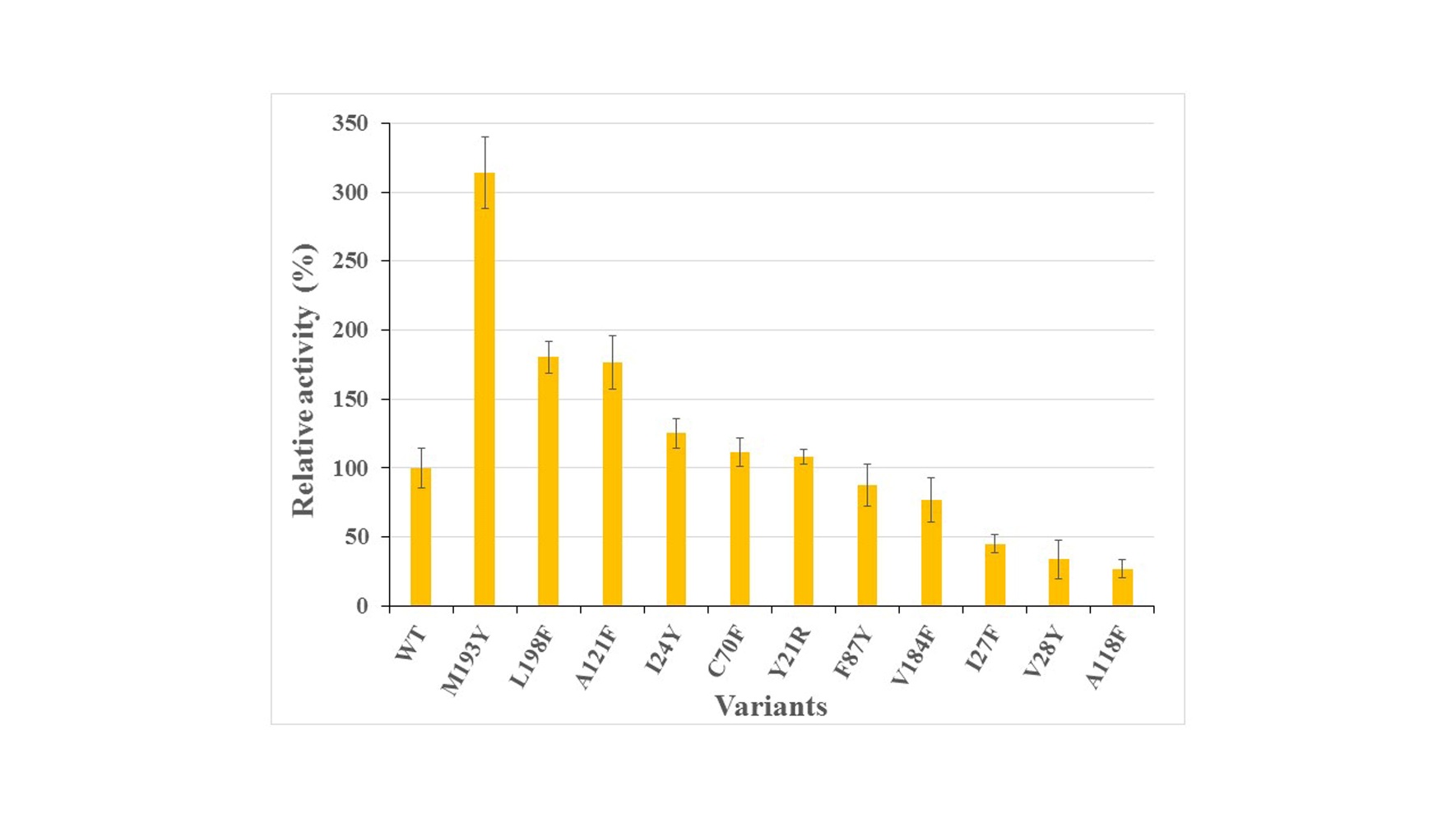


1. Relative activity of WT and variants towards *n*-nonal (A) and *n*-octanal (B)


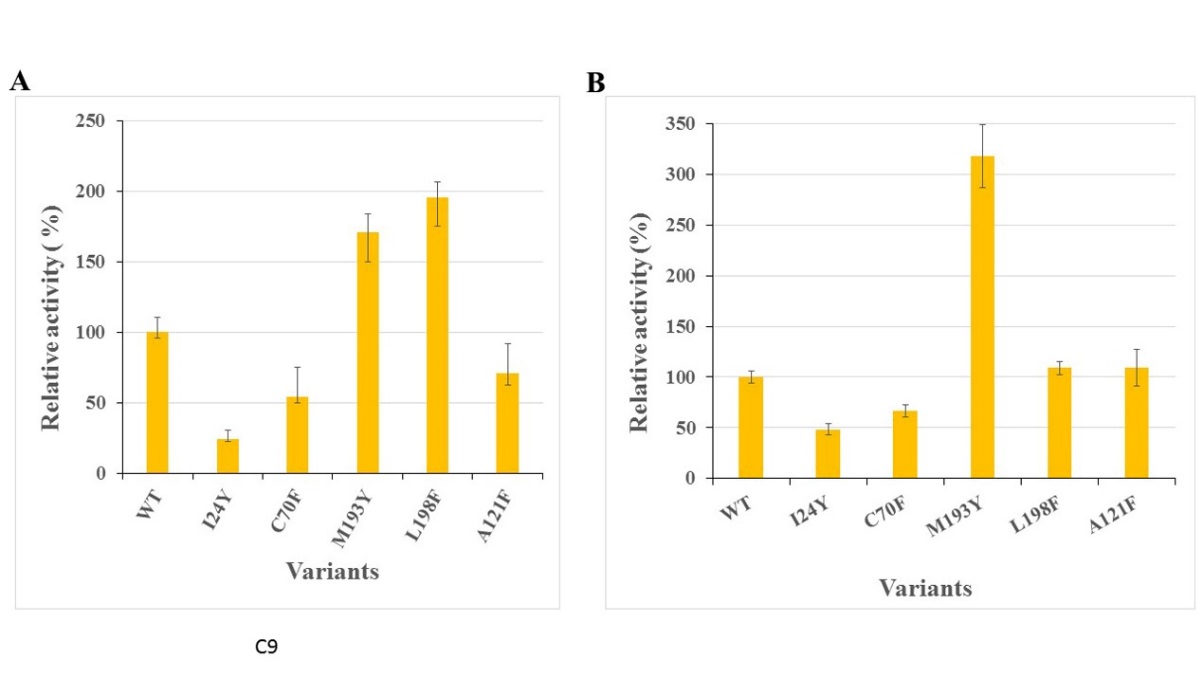


1. Relative activity of WT and variants towards *n*-hexanal (A) and *n*-butanal (B)


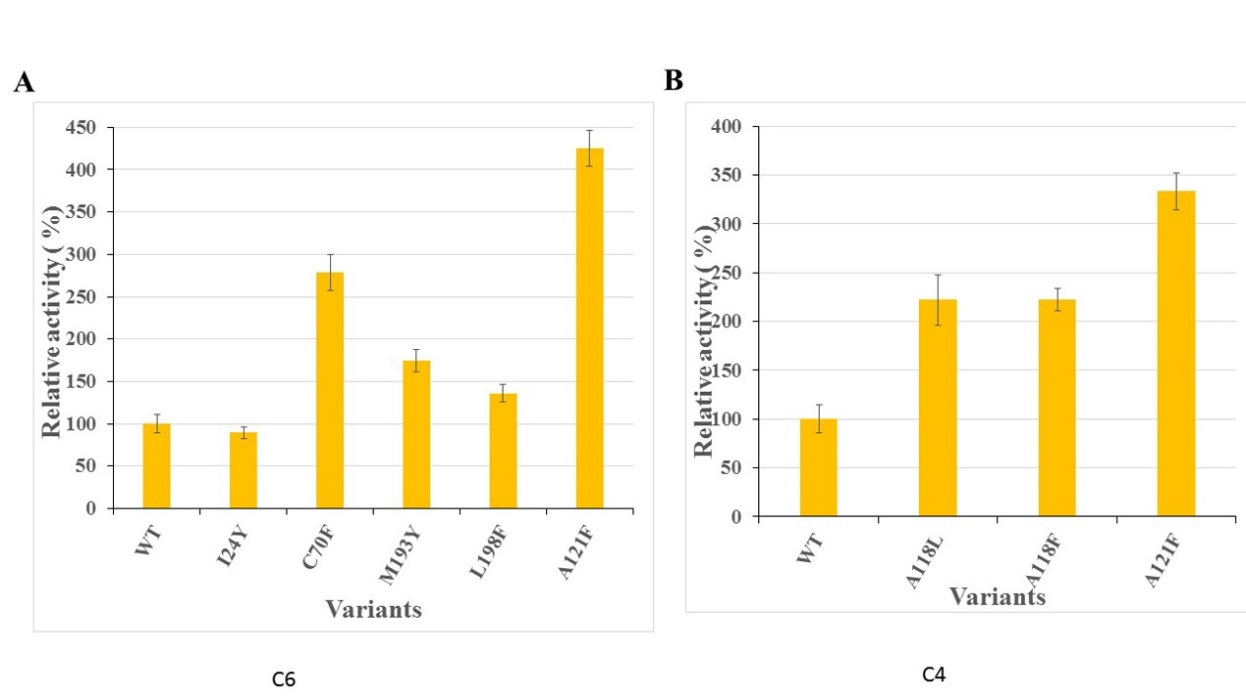

Supplement: Supplementary file 3 — 10.1186/s13068-016-0596-9 Relative activity of WT and cADO mutants against different substrates. [file 13068_2016_596_MOESM3_ESM.docx]
