# Supplementary material for: Structure-oriented substrate specificity engineering of aldehyde-deformylating oxygenase towards aldehydes carbon chain length
Source: Biotechnol Biofuels. 2016 Aug 31;9(1):185. doi: 10.1186/s13068-016-0596-9 (PMC5007808; doi:10.1186/s13068-016-0596-9)
Supplement: Supplementary file 4 — 10.1186/s13068-016-0596-9 Original data for determination of kinetic parameters of A121F and WT cADO against C6,7,8,9 aldehydes. [file 13068_2016_596_MOESM4_ESM.docx]

**Additional file 4**

**Original data for determination of kinetic parameters of A121F and WT cADO against C_6,7,8,9_ aldehydes**

**Based on Michaelis-Menten equation of GraphPad Prism 5**

1. **A121F-*n*-hexanal**

1. **A121F-*n*-heptanal**

1. **A121F-*n*-octanal**

1. **A121F-*n*-nonanal**

1. **WT-*n*-hexanal**

1. **WT-*n*-heptanal**

1. **WT-*n*-octanal**

1. **WT-*n*-nonanal**
