# Supplementary material for: Structure-oriented substrate specificity engineering of aldehyde-deformylating oxygenase towards aldehydes carbon chain length
Source: Biotechnol Biofuels. 2016 Aug 31;9(1):185. doi: 10.1186/s13068-016-0596-9 (PMC5007808; doi:10.1186/s13068-016-0596-9)
Supplement: Supplementary file 5 — 10.1186/s13068-016-0596-9 Secondary structure estimation based on CD spectra of WT and cADO variants. [file 13068_2016_596_MOESM5_ESM.docx]

**Additional file 5**

**Table S1 Secondary structure estimation based on CD spectra of WT and cADO variants**

Secondary structure were estimated with BeStSel method [36].

|  | **Secondary structure (%)** | | | | |
| --- | --- | --- | --- | --- | --- |
| Variants | α-Helix | Antiparallel β-strand | Parallel β-strand | Turn | Others |
| WT | 98.1 | 0.0 | 0.0 | 1.9 | 0.0 |
| L198F | 100.0 | 0.0 | 0.0 | 0.0 | 0.0 |
| M193Y | 95.0 | 0.0 | 0.0 | 5.0 | 0.0 |
| V184F | 98.6 | 0.0 | 0.0 | 1.4 | 0.0 |
| A121F | 99.9 | 0.0 | 0.0 | 0.1 | 0.0 |
| A118F | 95.6 | 0.5 | 0.0 | 3.9 | 0.0 |
| F87Y | 100.0 | 0.0 | 0.0 | 0.0 | 0.0 |
| C70F | 100.0 | 0.0 | 0.0 | 0.0 | 0.0 |
| V28Y | 100.0 | 0.0 | 0.0 | 0.0 | 0.0 |
| I27F | 94.4 | 0.0 | 0.0 | 5.6 | 0.0 |
| I24Y | 100.0 | 0.0 | 0.0 | 0.0 | 0.0 |
