# Supplementary material for: Structure-oriented substrate specificity engineering of aldehyde-deformylating oxygenase towards aldehydes carbon chain length
Source: Biotechnol Biofuels. 2016 Aug 31;9(1):185. doi: 10.1186/s13068-016-0596-9 (PMC5007808; doi:10.1186/s13068-016-0596-9)

**Additional file 6**

**Figure S3 Comparison of fatty alk(a/e)ne production in *E. coli* strains harboring WT cADO and V184F**


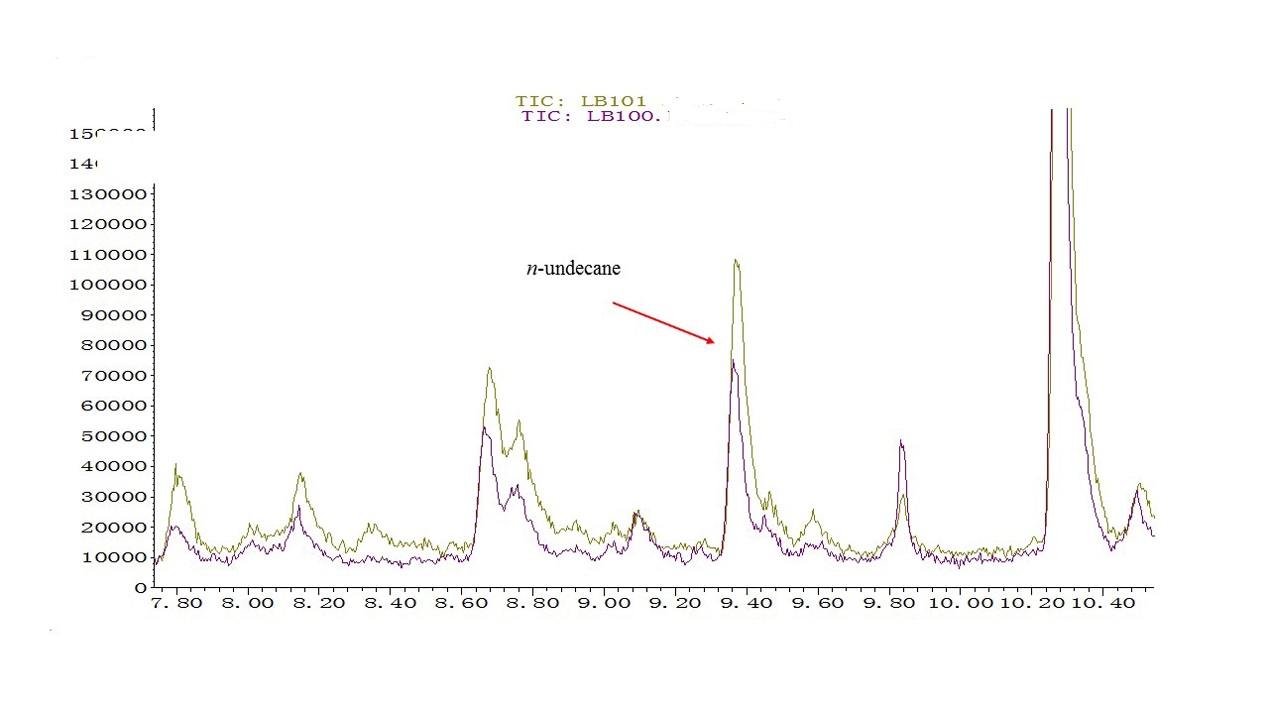


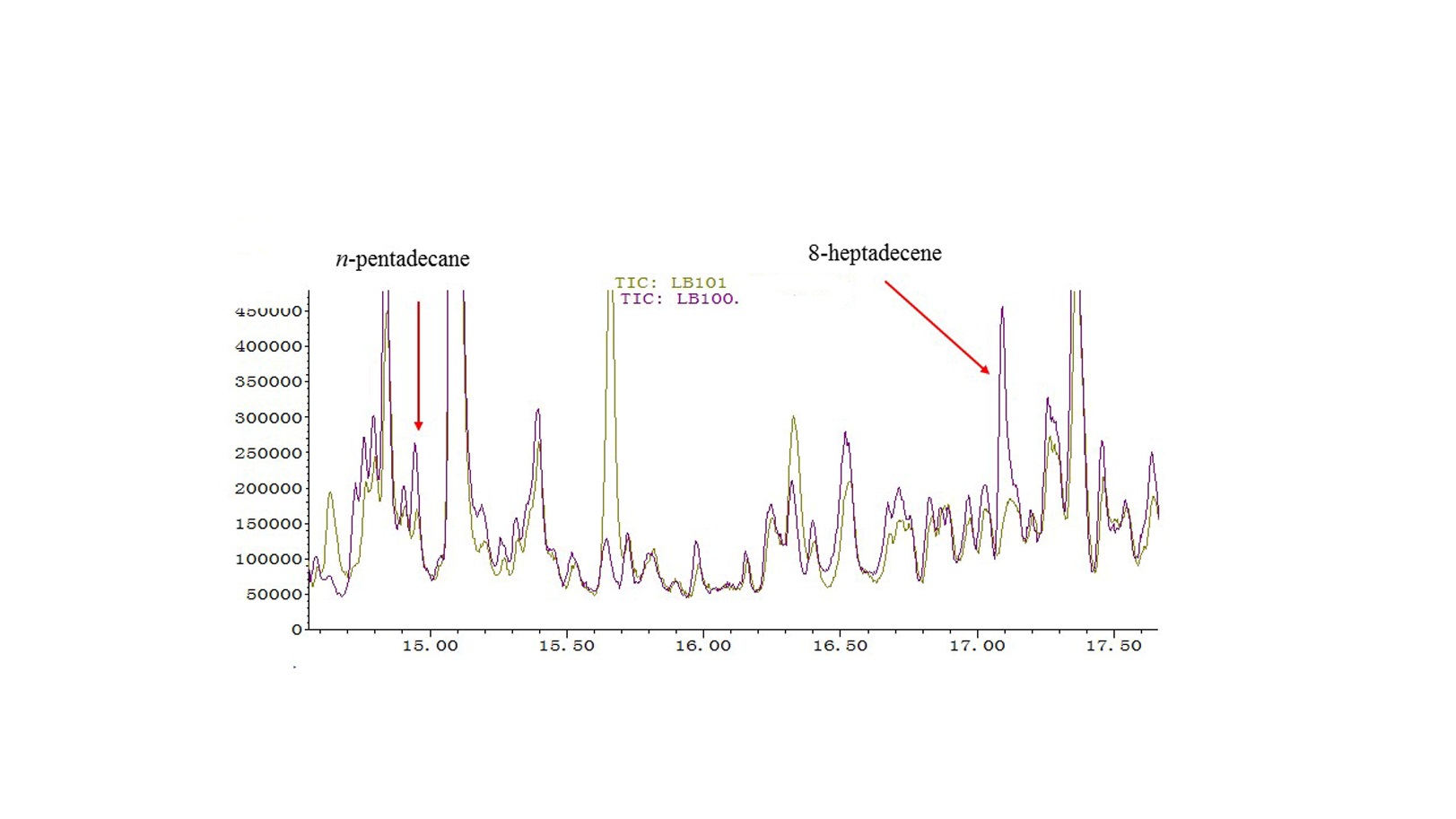

Supplement: Supplementary file 6 — 10.1186/s13068-016-0596-9 Comparison of fatty alk(a/e)ne production in E. coli strains harboring WT cADO and V184F. [file 13068_2016_596_MOESM6_ESM.docx]
