# Supplementary material for: Structure-oriented substrate specificity engineering of aldehyde-deformylating oxygenase towards aldehydes carbon chain length
Source: Biotechnol Biofuels. 2016 Aug 31;9(1):185. doi: 10.1186/s13068-016-0596-9 (PMC5007808; doi:10.1186/s13068-016-0596-9)

**Additional file 8**

**Figure S5 Part of superimposed structures of 1593 (PDB code: 4RC5) and PMT1231 (PDB code: 4PGI)**

Residue Ala194 of PMT1231, residues Tyr121 and Cys70 of 1593, and the bound substrate analogs were highlighted.


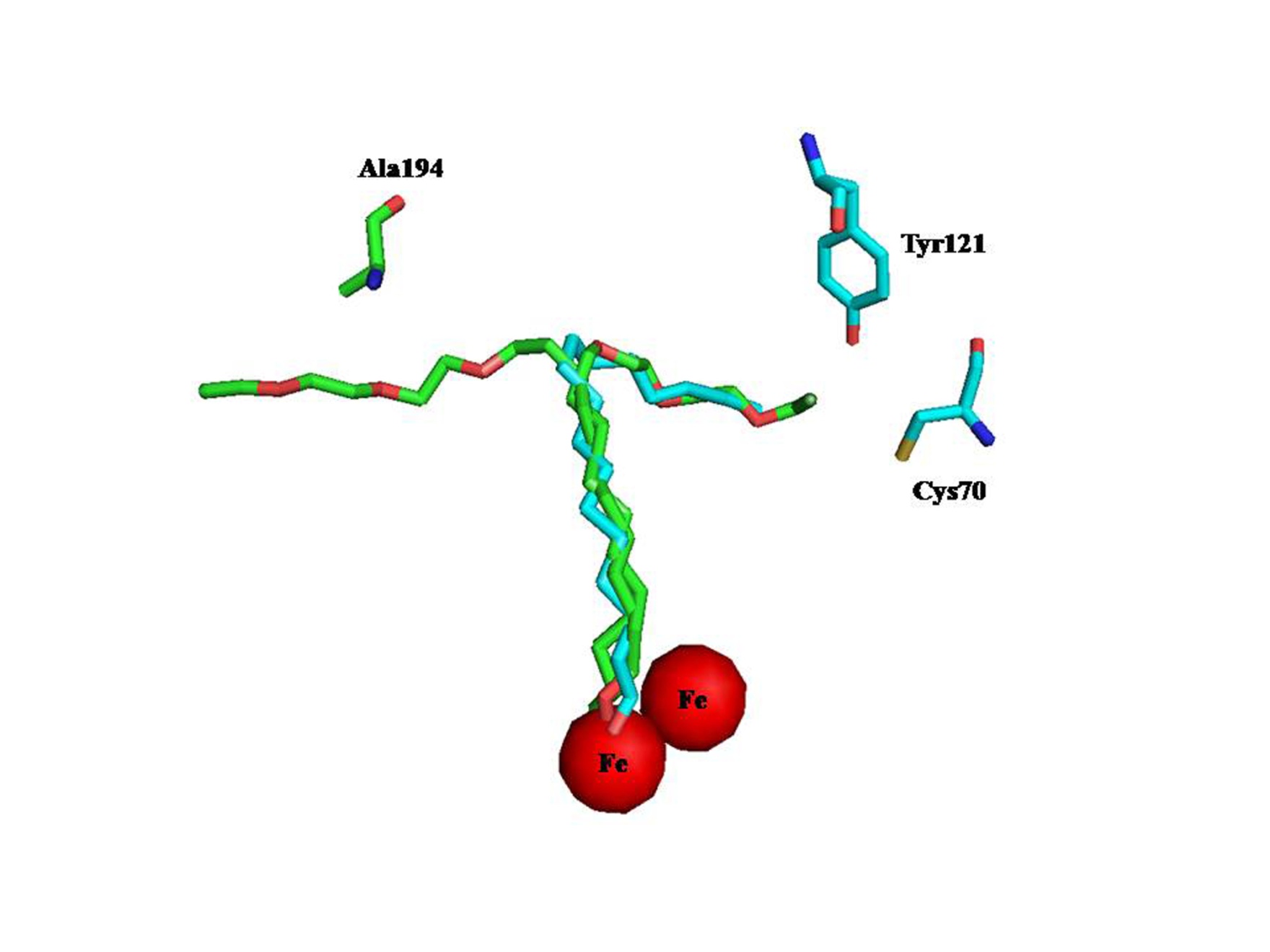

Supplement: Supplementary file 8 — 10.1186/s13068-016-0596-9 Part of superimposed structures of 1593 (PDB code: 4RC5) and PMT1231 (PDB code: 4PGI) [file 13068_2016_596_MOESM8_ESM.docx]
