# Supplementary material for: Structure-oriented substrate specificity engineering of aldehyde-deformylating oxygenase towards aldehydes carbon chain length
Source: Biotechnol Biofuels. 2016 Aug 31;9(1):185. doi: 10.1186/s13068-016-0596-9 (PMC5007808; doi:10.1186/s13068-016-0596-9)
Supplement: Supplementary file 9 — 10.1186/s13068-016-0596-9 Primers used for construction of site-directed mutants. [file 13068_2016_596_MOESM9_ESM.docx]

**Additional file 9**

**Table S2 Primers used for construction of site-directed mutants**

| Mutants | Primer sequence (5’→3’) |
| --- | --- |
| Y21R | For: CCTATAAAGACGCGAGGTCCCGCATCAACGCC |
|  | Rev: GGCGTTGATGCGGGACCTCGCGTCTTTATAGG |
| I24Y | For: GCGTACTCCCGCTACAACGCCATCGTGATTGAAG |
|  | Rev: CTTCAATCACGATGGCGTTGTAGCGGGAGTACGC |
| I27F | For: CTCCCGCATCAACGCCTTCGTGATTGAAGGT |
|  | Rev: ACCTTCAATCACGAAGGCGTTGATGCGGGAG |
| V28Y | For: GCATCAACGCCATCTACATTGAAGGTGAGC |
|  | Rev: CTGCTCACCTTCAATGTAGATGGCGTTGATGC |
| G31F | For: GCCATCGTGATTGAATTTGAGCAGGAAGCAT |
|  | Rev: ATGCTTCCTGCTCAAATTCAATCACGATGGCG |
| C70F | For: GGCTTCATGGCTTTCGGCAAGAATCTGAG |
|  | Rev: CAGATTCTTGCCGAAAGCCATGAAGCCTT |
| F87Y | For: TCGCGCAAAAATTCTACGAACGTCTGCAC |
|  | Rev: GTGCAGACGTTCGTAGAATTTTTGCGCGA |
| A118F | For: TATCGAGTGCTTCTTCATCGCTGCATACAAC |
|  | Rev: TGTATGCAGCGATGAAGAAGCACTCGATAAT |
| A118L | For: GATTATCGAGTGCTTCCTGATCGCTGCATACAAC |
|  | Rev: GTTGTATGCAGCGATCAGGAAGCACTCGATAATC |
| A121F | For: GAGTGCTTCGCTATCGCTTTCTACAACATCTACATC |
|  | Rev: GATGTAGATGTTGTAGAAAGCGATAGCGAAGCACTC |
| V184F | For: GATGCTGAACGAATTTGCAGACGACGCTC |
|  | Rev: GAGCGTCGTCTGCAAATTCGTTCAGCATC |
| M193Y | For: CGCGAACTGGGCTACGAACGTGAATCTCTG |
|  | Rev: CAGAGATTCACGTTCGTAGCCCAGTTCGCG |
| L198F | For: GGCATGGAACGTGAATCTTTCGTTGAAGACTTC |
|  | Rev: GAAGTCTTCAACGAAAGATTCACGTTCCATGCC |
